# The Non‐Coding Regulatory Variant rs2863002 at chr11p11.2 Increases Neuroblastoma Risk by Affecting *HSD17B12* Expression and Lipid Metabolism

**DOI:** 10.1002/advs.202415181

**Published:** 2025-06-17

**Authors:** Teresa Maiorino, Marianna Avitabile, Vincenzo Aievola, Annalaura Montella, Vito A. Lasorsa, Ferdinando Bonfiglio, Mariagrazia Cantalupo, Sueva Cantalupo, Gilda Estinto, Matilde Tirelli, Martina Morini, Martina Ardito, Alessandra Eva, Vincenza Cerbone, Lucia Mauriello, Marianna Caterino, Margherita Ruoppolo, John M. Maris, Sharon J. Diskin, Achille Iolascon, Mario Capasso

**Affiliations:** ^1^ Department of Molecular Medicine and Medical Biotechnology at University of Naples “Federico II” Naples 80131 Italy; ^2^ CEINGE Biotecnologie Avanzate Franco Salvatore Naples 80145 Italy; ^3^ Department of Electrical Engineering and Information Technology at University of Naples “Federico II” Naples 80125 Italy; ^4^ Laboratory of Experimental Therapies in Oncology IRCCS Istituto Giannina Gaslini Genoa 16147 Italy; ^5^ Scientific Directorate IRCCS Istituto Giannina Gaslini Genoa 16147 Italy; ^6^ The Children’s Hospital of Philadelphia and the Department of Pediatrics Perelman School of Medicine University of Pennsylvania Philadelphia PA 19104 USA

**Keywords:** functional genomics, genetic predisposition, GWAS, *HSD17B12*, lipid metabolism, neuroblastoma, SNP

## Abstract

A Genome‐wide association study (GWAS) on a European‐American cohort identified chr11p11.2 as a neuroblastoma predisposition locus. Combining in‐house and public genomic data from neuroblastoma cell lines, this work implicates rs2863002 as the candidate causal variant at the 11p11.2 locus, confirming its cis‐regulatory activity through a luciferase reporter assay. The genetic association of rs2863002 with neuroblastoma risk is validated in an Italian case‐control cohort. Using ChIP‐qPCR, Hi‐C, and CRISPR genome editing, this work deciphers the regulatory mechanisms at the risk locus, demonstrating that the rs2863002‐C risk allele regulates *HSD17B12* expression and reduces GATA3 binding affinity. In vitro functional assays and targeted lipidomic analyses reveal the involvement of the rs2863002‐C risk allele in tumorigenicity and modulation of lipid metabolism in neuroblastoma cells through *HSD17B12* regulation. This study provides new insights into the genetic basis of neuroblastoma and underscores the importance of post‐GWAS functional characterization of risk loci in uncovering relevant biological findings for understanding complex diseases.

## Graphical abstract

The SNP rs2863002 at the 11p11.2 locus, identified through GWAS, is associated with an increased risk of neuroblastoma. The rs2863002‐C allele impairs the binding of the transcription factor GATA3, resulting in upregulation of the *HSD17B12* gene. Overexpression of *HSD17B12* promotes a pro‐tumorigenic phenotype in neuroblastoma cells and leads to alterations in lipid metabolism.

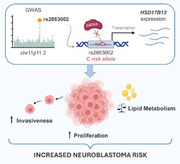

## 1 Introduction

Recent advancements in large‐scale genomic analyses have significantly broadened our understanding of the genetic underpinnings of childhood cancers. Neuroblastoma, the most common extracranial solid tumor in infants, arises from the malignant transformation of neural crest cell precursors and affects ≈1 in 7000 children, primarily those under the age of 5.^[^ ^1^ ^]^ Unlike adult cancers, which often exhibit complex genetic profiles and high somatic mutation rates, pediatric tumors like neuroblastoma typically show fewer somatic mutations and a greater proportion of germline alterations.^[^ ^2^ ^]^ Notably, a substantial percentage of children with cancer carry germline genetic variants that predispose them to the disease.^[^ ^3^, ^4^, ^5^, ^6^ ^]^

The clinical behavior of neuroblastoma varies remarkably, ranging from spontaneous regression to aggressive high‐risk forms.^[^ ^1^ ^]^ Several genomic markers are crucial for categorizing neuroblastoma risk. The amplification of the *MYCN* oncogene is often associated with poor prognosis.^[^ ^7^ ^]^ Other significant chromosomal aberrations include deletions of 1p and 11q, gains of 17q, rearrangements at the *TERT* locus, and the combination of 19p loss with 1q gain in older children.^[^ ^7^, ^8^ ^]^ Despite the identification of clinically actionable alterations, including those in the *ALK* and *ATRX* genes, and the *FGFR1* gene mutation recently reported, the 5‐year survival rate for high‐risk patients remains unfortunately low.^[^ ^9^, ^10^ ^]^

Genome‐wide Association Studies (GWASs) have emerged as a powerful tool to investigate the genetic etiology of neuroblastoma. These studies have led to the identification of common single nucleotide polymorphisms (SNPs) associated with disease risk. To date, GWAS loci and associated susceptibility genes include *CASC15*, *BARD1*, *LMO1*, *DUSP12*, *HSD17B12*, *DDX4/IL31RA*, *HACE1*, *LIN28B*, *TP53*, *MLF1*, *CPZ*, *CDKN1B*, *NEFL*, *SLC16A1*, *FEN1* and *ALKBH5*.^[^ ^11^, ^12^, ^13^, ^14^ ^]^ These genetic variants generally reside in the non‐coding portion of the genome and may have regulatory effects that impact cancer susceptibility.

While GWASs facilitate the initial identification of risk loci, distinguishing the true causal association variants that contribute to the genetic predisposition can be challenging. Relevant common risk variants could be concealed by linkage disequilibrium (LD) or hidden within signals discarded by restrictive analytical steps, such as multiple testing corrections.^[^ ^15^ ^]^ To address these limitations, post‐GWAS functional characterization techniques and variant‐to‐function analyses have become crucial. These approaches bridge the gap between statistical associations linking loci to traits and the underlying biological mechanisms of disease risk variants.^[^ ^16^, ^17^, ^18^ ^]^

Functional analyses of neuroblastoma‐associated loci uncovered through GWASs have shed light on the molecular mechanisms driving neuroblastoma pathogenesis. For instance, studies of the 6p22 neuroblastoma risk locus revealed that the long non‐coding RNA CASC15 plays a role in the onset and progression of neuroblastoma.^[^ ^19^, ^20^, ^21^ ^]^ Similarly, after identifying common variants in *BARD1* associated with high‐risk neuroblastoma,^[^ ^22^ ^]^ subsequent research elucidated its significant function as a tumor suppressor gene, particularly in DNA repair pathways.^[^ ^23^, ^24^, ^25^ ^]^ Another relevant example is the discovery of *LIN28B* as a neuroblastoma susceptibility gene,^[^ ^26^ ^]^ later demonstrated to be a key player in oncogenic signaling and neuroblastoma progression.^[^ ^27^, ^28^ ^]^

Despite these advances, some genome‐wide significant neuroblastoma‐associated loci, such as the chr11p11.2 locus,^[^ ^22^, ^29^ ^]^ have not yet undergone functional investigation. Our study addresses this gap by focusing on this locus. Through a combination of in silico analyses, in vitro experiments, and functional genomics approaches, we identified the SNP rs2863002 as a regulatory variant within *HSD17B12*, a gene implicated in various cancer types.^[^ ^30^, ^31^, ^32^ ^]^ Our comprehensive investigation reveals how this variant influences *HSD17B12* expression, thereby impacting lipid metabolism and oncogenesis in neuroblastoma.

## 2 Results

### 2.1 The Variant rs2863002 at the Neuroblastoma Risk Locus chr11p11.2 Shows Regulatory Functions

**Figure 1:**
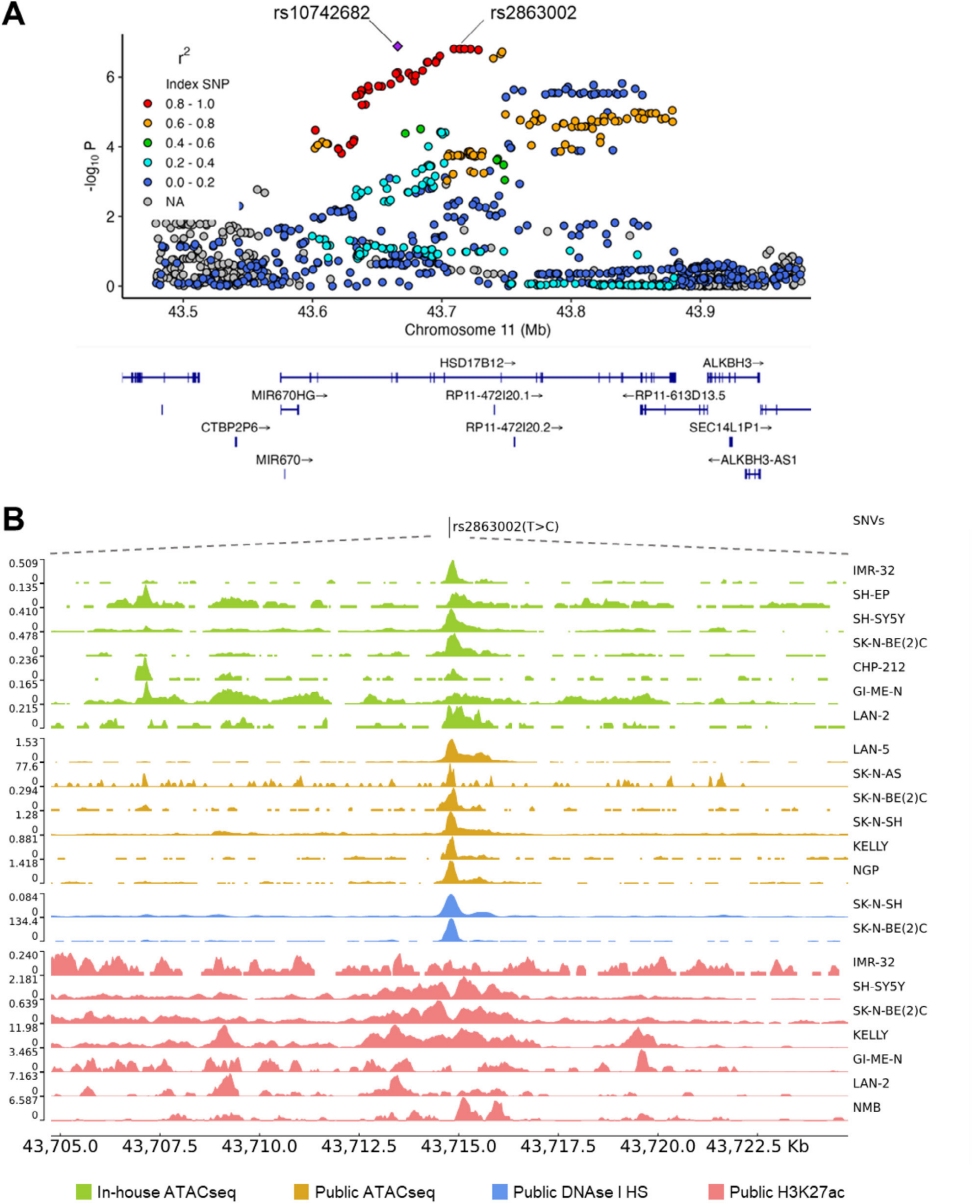
rs2863002 is the functional regulatory SNP of the 11p11.2 neuroblastoma predisposition locus. A) Regional association plot of genotyped SNPs at the 11p11.2 neuroblastoma susceptibility locus implicating *HSD17B12*. Y‐axis represents the significance of association (‐log10 transformed *p* values), while the X‐axis shows the genomic location on chromosome 11 (Mb). SNPs are color‐coded based on pair‐wise linkage disequilibrium (r^2^) with the most statistically significant SNP rs10742682 (top violet triangle) (*p*‐value = 1.31 × 10^−7^, OR: 1.24, 95% CI: 1.15–1.34). Plot generated using LocusZoom. B) The image shows a narrowed‐down region spanning 10 kb up‐ and down‐stream of rs2863002 (chr11:43 714 768, hg19/GRCh37). The peak‐based tracks represent, from top to bottom, the in‐house generated (green) and public (yellow) ATAC‐seq profiles of human neuroblastoma cell lines, the DNase‐I hypersensitivity levels (light blue), and the H3K27ac ChIP‐seq (pink) density profiles obtained from the NCBI GEO public database. Data ranges are shown on the left of each track, while neuroblastoma cell lines are reported on the right.

The latest neuroblastoma GWAS, which included 2101 European‐American cases and 4202 controls, identified the genetic variant rs10742682 at the chr11p11.2 risk locus within the *HSD17B12* gene as the most significant SNP associated with neuroblastoma risk (*p*‐value = 1.31 × 10^−7^; OR = 1.24, 95% CI: 1.15–1.34).^[^ ^29^ ^]^ To identify SNPs with functional regulatory effects, we selected 42 SNPs within the chr11p11.2 locus that were in linkage disequilibrium (LD) (r^2^>0.8) with the index SNP rs10742682 (**Figure** **1**A). These SNPs were annotated using newly generated ATAC‐seq data from 9 neuroblastoma cell lines, as well as public ATAC‐seq and H3K27ac ChIP‐seq data from 44 human neuroblastoma cell lines available in the GEO database (Tables S1 and S2 and Figure S1, Supporting Information). Additionally, transcription factor (TF) ChIP‐seq data were used to predict if these SNPs map inside TF binding sites and their capacity to bind TFs with biological relevance in neuroblastoma (Tables S1 and S2 and Figure S1, Supporting Information). Among the 42 variants examined, beyond the lead SNP rs10742682 (chr11:43 665 857, hg19 GRCh37) and the closely positioned rs10742681 (chr11:43 665 856, hg19 GRCh37), we identified the rs2863002 variant as particularly noteworthy. This variant was found to be located in open chromatin regions identified in 29 ATAC‐seq and 5 H3K27ac ChIP‐seq experiments in various neuroblastoma cell lines (Figure 1B, Table S1, and Figure S1, Supporting Information), but also showed the highest enrichment of TF binding sites, including MYCN, LMO1, GATA3, HAND2, ISL1, PHOX2B, and TBX2 (Table S1 and Figure S1, Supporting Information). The abundance of binding sites for TF with relevant roles in neuroblastoma implies significant transcriptional regulation in this region, suggesting that the rs2863002 SNP could either disrupt or create new TF binding sites.

Moreover, to add an extra layer of significance to our analysis, we applied a functionally‐informed fine‐mapping strategy using PolyFun in combination with the SuSiE algorithm.^[^ ^33^ ^]^ This method integrates a comprehensive set of functional annotations, including coding, conserved, regulatory and LD‐related features, derived from ≈19 million imputed SNPs in the UK Biobank dataset (MAF > 0.1%). Within this framework, the variant rs2863002 ranked among the top three SNPs in the 95% credible set, which denotes the subset of variants that collectively harbor a 95% posterior probability of including the causal variant (Table S3, Supporting Information). This finding provides additional and independent support to the robustness and biological relevance of our initial prioritization strategy.

Therefore, we selected rs2863002 T>C, located within the first intron of *HSD17B12*, as the most probable candidate functional variant at the chr11p11.2 risk locus. The minor allele C was associated with an increased risk of neuroblastoma development (*p*‐value = 1.56 × 10^−7^; OR = 1.23, 95% CI: 1.14–1.33) in GWAS. We validated this genetic association by performing a PCR‐based genotyping in an independent cohort of 607 neuroblastoma cases and 2032 controls with Italian origins (*p*‐value = 6.51 × 10^−4^, OR = 1.28, CI: 1.11–1.48) (Tables S4 and S5, Supporting Information). Additionally, we carried out an inverse variance‐weighted meta‐analysis to combine data from the validation cohort with those from the initial neuroblastoma GWAS. The candidate SNP rs2863002 achieved significant association with neuroblastoma risk at a genome‐wide level (*p*‐value = 4.36 × 10^−10^, OR = 1.25, CI: 1.16–1.34) in the comprehensive meta‐analysis (**Table** **1**).

**Table 1:** Meta‐analysis of the genetic association between rs2863002 SNP and neuroblastoma risk.

SNP	Major Allele	Minor Allele	Cohort	Minor Allele Frequency Cases	Minor Allele Frequency Controls	^b)^ *p*‐value	OR (95% CI)
rs2863002	T	C	^a)^European American	0.46 (N = 2101)	0.41 (N = 4202)	1.56 × 10^−7^	1.23 (1.14–1.33)
			Italian	0.41 (N = 607)	0.35 (N = 2032)	6.51 × 10^−4^	1.28 (1.11–1.48)
			Combined			4.36 × 10^−10^	1.25 (1.16–1.34)

^a)^ Data obtained by McDaniel et., 2017;

^b)^ *p*‐value calculated using logistic regression, with age and sex as covariates; OR: odd ratio; CI: confidence interval.

Our findings suggest the role of rs2863002 as the functional SNP at the chr11p11.2 neuroblastoma susceptibility locus, showing that it is located inside a putative enhancer regulatory element in neuroblastoma cell lines.

### 2.2 rs2863002 Exerts its Regulatory Activity by Altering a GATA3 Binding Site

Given that the rs2863002 variant is situated in a region capable of binding several neuroblastoma‐relevant TFs, we performed an in silico motif enrichment analysis to identify TFs with enriched binding motifs within a 50 bp region centered on this functional SNP. Using a database of human TFs that includes both activators and repressors of transcription, our analysis revealed multiple significantly enriched TF motifs overlapping the genomic position of rs2863002 (Table S6 and Figure S2, Supporting Information).

**Figure 2:**
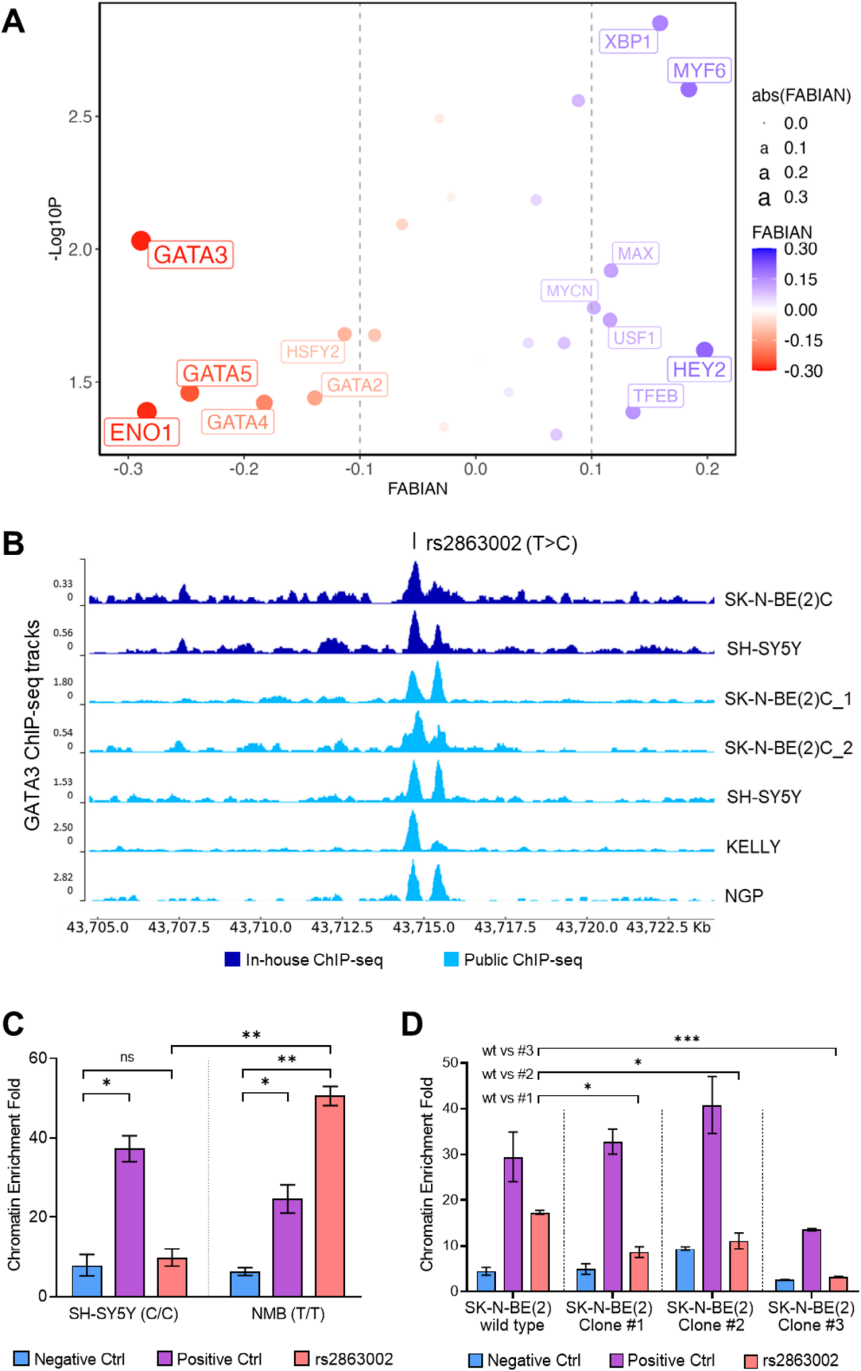
The rs2863002 SNP alters the binding site for the transcription factor GATA3. A) The graph shows the combined results of the TF motifs enrichment analysis (‐Log10P, y‐axis) and the FABIAN scores related to the alteration of the TF binding motifs due to rs2863002 (FABIAN, x‐axis). Each dot represents a TF binding motif with color and size related to the score obtained with the FABIAN prediction tool, while the dotted lines represent the threshold values chosen to classify TF motif disruption (red) or gain (blue). B) ChIP‐seq tracks for GATA3 TF obtained from neuroblastoma cell lines. The image shows rs2863002 at chr11:43 714 768 (hg19/GRCh37) in correspondence with in‐house generated (dark blue) and publicly available (light blue) GATA3 ChIP‐seq data from neuroblastoma cell lines deposited in the GEO database. Data ranges are shown on the left, while neuroblastoma cell lines are reported on the right. C) Chromatin fold enrichment obtained in GATA3 ChIP qPCR experiments carried out in SH‐SY5Y and NMB cells. We report the chromatin fold enrichment obtained for a negative (chr5:24682868–24682979) and a positive (chr2:15982438–15982518) control region for GATA3 binding, respectively in blue and violet, and for the genomic region of rs2863002 in pink. Enrichment measurements are folded on Rabbit IgG and represent the mean ±SD from three independent experiments. D) Chromatin fold enrichment obtained in GATA3 ChIP qPCR experiments carried out in SK‐N‐BE(2) wild‐type cells and relative CRISPR/cas9 edited clones (clone#1, #2, and #3). We report the chromatin fold enrichment obtained for a negative and a positive DNA control region for GATA3 binding, and for the genomic region of rs2863002, as in (B). ns not significant; * *p*‐value < 0.05; ** *p*‐value < 0.01; *** *p*‐value < 0.001. *p*‐values were calculated by *t*‐test.

Concurrently, we employed the FABIAN tool to predict which TFs' binding affinities could be specifically altered by the two alleles of rs2863002—the protective T allele and the risk C allele (Table S7, Supporting Information). By integrating the results obtained from the motif enrichment analysis and the FABIAN predictions, we identified GATA3 as the top candidate TF whose binding affinity could be modulated by the allelic variation at rs2863002. Notably, GATA3 exhibited a highly significant enrichment of its binding motif (*p*‐value = 0.009) and showed the greatest predicted alteration in binding affinity due to the SNP among all TFs analyzed (FABIAN score = −0.289) (**Figure** **2**A).

The analysis of public ChIP‐seq data confirmed that GATA3 binds to the genomic region containing rs2863002 in 5 different neuroblastoma cell lines (Figure 2B). In particular, the FABIAN analysis predicted a decreased affinity of the GATA3 binding site in the presence of the rs2863002 C risk allele. To validate this prediction, we performed in vitro ChIP‐qPCR assays in SH‐SY5Y and NMB neuroblastoma cell lines, which harbor the C/C and T/T rs2863002 genotypes, respectively. The results showed reduced GATA3 binding in the region containing the rs2863002 SNP in the SH‐SY5Y cell line (C/C genotype) compared to the NMB cell line (T/T genotype) (Figure 2C). This finding confirms that the rs2863002‐C allele alters the GATA consensus binding motif, thereby reducing GATA3 binding affinity.

Interestingly, our combined predictive analysis of TF motifs altered by rs2863002 suggested a potential binding gain for MYCN, albeit with lower significance (Figure 2A). To further investigate this observation, we conducted ChIP‐qPCR experiments to assess MYCN differential binding to the two rs2863002 alleles under the same conditions described above. However, the results showed no significant binding of MYCN to the region containing the functional SNP, nor any differential binding affinity between the two rs2863002 alleles (Figure S3, Supporting Information).

In further exploration of the rs2863002 regulatory mechanism, we employed the CRISPR/Cas9 genome editing technique to introduce INDELs (insertions and deletions) around the SNP region. To this aim, a single guide RNA (sgRNA) was designed to target the region encompassing rs2863002. However, successful genome editing was not achievable in SH‐SY5Y cell lines with the homozygous C/C genotype (Figure S4A, Supporting Information) because the C allele disrupted the sgRNA's target alignment, significantly diminishing Cas9 cutting efficiency. Additionally, NMB cell lines with the homozygous T/T genotype (Figure S4A, Supporting Information) were unsuitable for single‐cell cloning, complicating the isolation of individual clones. Consequently, we opted to perform our genome editing experiments in the SK‐N‐BE(2) neuroblastoma cell line, which is heterozygous for the rs2863002 (T/C) variant (Figure S4A, Supporting Information) and has been previously validated for CRISPR/Cas9 assays.^[^ ^34^ ^]^ Three clones were chosen for further analysis: one exhibited a 20 bp deletion encompassing the rs2863002 site, and the other two showed a 1 bp insertion immediately upstream of the SNP (Figure S4B,C, Supporting Information).

We tested the GATA3 TF binding affinity in the CRISPR/Cas9 edited cells and observed that the alteration of the T allele in the edited clones induced a reduction in the binding enrichment for GATA3 compared to the SK‐N‐BE(2) wild‐type cell line (Figure 2D). This finding aligns with our initial prediction regarding GATA3 binding at the rs2863002 genomic position.

Collectively, these results demonstrate that GATA3 is involved in the regulatory mechanism at the chr11p11.2 locus and that the rs2863002‐C risk allele likely impairs the GATA3 consensus motif sequence and its binding capacity.

### 2.3 *HSD17B12* is the Target Gene of rs2863002 Regulatory Activity at the chr11p11.2 Neuroblastoma Risk Locus

**Figure 3:**
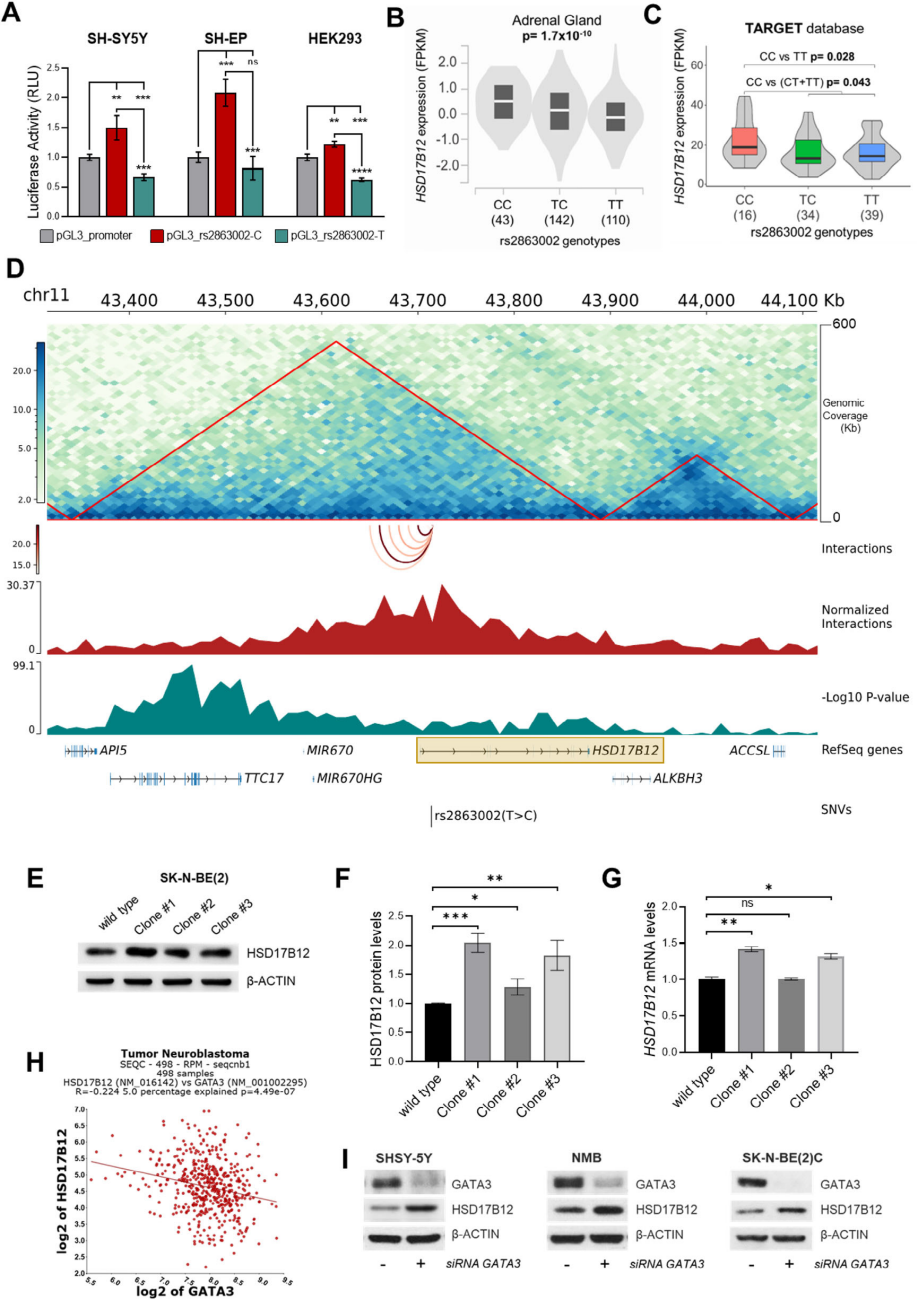
The rs2863002 SNP acts as an enhancer in neuroblastoma cells, positively influencing *HSD17B12* expression. A) Luciferase reporter gene assays were carried out in the SH‐SY5Y, SH‐EP, and HEK293 cell lines. Luciferase activity of the constructs harboring the C and T alleles of rs2863002 was compared to a PGL3 empty control vector. The results are expressed as relative luminescence units (RLU) and the ratio between firefly/renilla luciferases provided the normalized luciferase activity for each vector. Data represent the mean ± SD of three independent experiments and *p*‐values were obtained by *t*‐test. B,C) Violin plots showing the median expression of *HSD17B12* according to rs2863002 genotypes in adrenal gland tissue (GTEx portal) (B), and in the TARGET database of neuroblastoma patients (C). D) Hi‐C results obtained in SK‐N‐BE(2)C neuroblastoma cell line, showing the genomic region including rs2863002 on genome assembly hg19/GRCh37. The interaction matrix is centered on rs2863002 at chr11:43 714 768 and extended of 0.4 Mb up‐ and down‐stream. Genomic coverage is 500Kb and the matrix resolution is 10Kb. Red triangles represent the Topologically Associated Domains (TADs). The genomic tracks displayed from top to bottom are: the arcs track showing the interactions between rs2863002 and the up‐stream annotated bins; the normalized number of interactions; the minus Log10 of the FDR adjusted *p*‐values; the NCBI RefSeq genes. A brown‐bordered rectangle highlights the *HSD17B12* locus. E) Representative western blot image of HSD17B12 expression in SK‐N‐BE(2) wild‐type and CRISPR/Cas9‐edited clones. β‐Actin protein level was used as the loading control. F,G) Quantitative measurements of HSD17B12 protein (F) and mRNA (G) expression in SK‐N‐BE(2) wild‐type and CRISPR/Cas9‐edited clones. H) Correlation analysis of *HSD17B12* and *GATA3* expression from R2 Genomics (GSE62564). R, correlation coefficient; P, *p*‐value. I) Western blot images of GATA3 and HSD17B12 protein levels after 72 h of GATA3 siRNA transfection in SH‐SY5Y, NMB, and SK‐N‐BE(2)C. ns non‐significant; * *p*‐value < 0.05; ** *p*‐value < 0.01; *** *p*‐value < 0.001. *p*‐values were calculated by *t*‐test.

To investigate the functional potential of rs2863002 and determine whether this SNP can influence the activity of the enhancer element where it resides, we conducted in vitro luciferase reporter assays in two neuroblastoma cell lines, SH‐SY5Y and SH‐EP, and in the non‐neuroblastoma cell line HEK293. Our findings demonstrated that the rs2863002‐C risk allele significantly enhanced the transcriptional activity of the reporter gene when compared to the rs2863002‐T protective allele (**Figure** **3**A), thus confirming that the rs2863002‐C allele functions as an enhancer element in neuroblastoma.

Subsequently, to examine the effect of rs2863002 on gene expression, we conducted cis‐expression Quantitative Trait Loci (eQTL) analysis using data from the adrenal gland tissue —the primary site of neuroblastoma onset— sourced from the GTEx portal. This analysis revealed that rs2863002 significantly influences the expression of *HSD17B12*, the gene within which the variant is located, with a *p*‐value of 1.7 × 10^−10^ (Figure 3B). Notably, while we acknowledge the possibility of other target genes, in our eQTL investigation *HSD17B12* was the only protein‐coding gene whose expression was significantly associated with the SNP regulatory activity. Specifically, individuals with the C/C genotype of rs2863002 exhibited higher *HSD17B12* expression levels (Figure 3B). Consistent with these findings, an analysis of Whole Genome Sequencing (WGS) and RNA‐seq data from 89 neuroblastoma patients from the TARGET project suggested a similar correlation (Figure 3C).

The regulatory potential of the SNP on *HSD17B12* expression was further confirmed by significant chromatin interactions between the *HSD17B12* promoter and the enhancer region containing rs2863002, observed both in our Hi‐C data in the SK‐N‐BE(2)C neuroblastoma cell line (Figure 3D and Table S8, Supporting Information) and in the publicly available Hi‐C data from the SK‐N‐DZ neuroblastoma cell line (Figure S5, Supporting Information).

To ultimately confirm the direct link between the regulatory activity of rs2863002 at chr11p11.2 locus and *HSD17B12*, we tested the expression of the target gene in the edited clones obtained through the CRISPR/Cas9 genome editing experiments presented above. Irrespective of the editing event, modifications near or at rs2863002 led to upregulated *HSD17B12* expression at both the protein and mRNA levels (Figure 3E–G and Figure S6, Supporting Information), indicating that the C risk allele of rs2863002 enhances *HSD17B12* expression.

Moreover, to assess GATA3 role in the regulation of *HSD17B12* expression mediated by rs2863002, we analyzed RNA‐seq data from 498 neuroblastoma tumors (SEQC database, GSE62564) and revealed an inverse correlation between the expression of *GATA3* and *HSD17B12* (R‐value = −0.224; *p*‐value = 4.49 × 10^−7^), thus assuming that in the presence of the rs2863002‐C allele the reduction of GATA3 binding leads to increased *HSD17B12* expression (Figure 3H). Additionally, we silenced *GATA3* by siRNA transient transfection in neuroblastoma cell lines to mimic GATA3 binding impairment, and observed a concomitant increase in *HSD17B12* expression (Figure 3I), thereby confirming the negative regulatory relationship and indicating that GATA3 functions as a transcriptional repressor for *HSD17B12* expression. Altogether, these results demonstrate that *HSD17B12* represents the target gene whose expression is modulated by the functional SNP rs2863002 at the chr11p11.2 risk locus and bolster the hypothesis that GATA3 plays a critical role in this regulatory mechanism of gene expression.

### 2.4 The Functional SNP rs2863002 Modulates Neuroblastoma Cell Tumorigenicity through the Regulation of *HSD17B12* Expression

In exploring the potential oncogenic role of *HSD17B12* in neuroblastoma, we examined its expression in the context of prognostic factors using the extensive RNA‐seq database SEQC (GSE62564), encompassing 498 neuroblastoma tumor samples. Our analysis revealed that high *HSD17B12* expression is significantly associated with adverse clinical indicators, such as stage 4 disease (Stage 4 versus Stage 1: *p*‐value = 3 × 10^−5^), *MYCN* amplification (*p*‐value = 4.14 × 10^−7^), and classification within the high‐risk neuroblastoma group (*p*‐value = 3.25 × 10^−11^) (Figure S7A–C, Supporting Information). Moreover, high *HSD17B12* expression was associated with poorer event‐free (*p*‐value = 2 × 10^−8^) and overall (*p*‐value = 1 × 10^−7^) survival rates (Figure S7D,E, Supporting Information). Cox regression analysis proved that elevated *HSD17B12* expression is a standalone predictor of poorer survival outcomes, irrespective of other prognostic disease markers, demonstrating a particularly strong relationship with event‐free survival (Table S9, Supporting Information). Altogether, these findings suggest that the gene could function as an oncogene in neuroblastoma.

**Figure 4:**
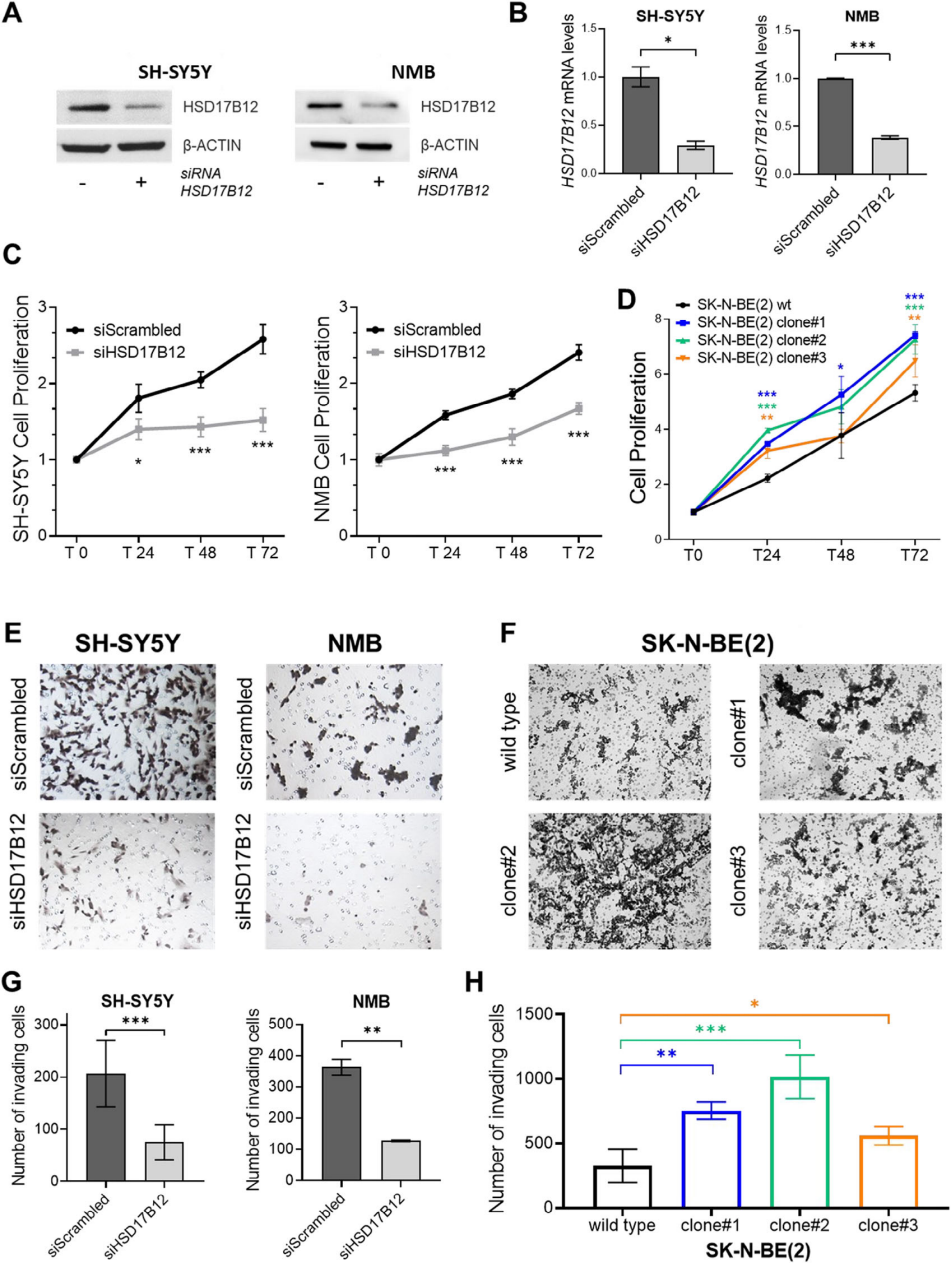
*HSD17B12* is an oncogenic driver enhancing cell growth and invasion in neuroblastoma. A,B) *HSD17B12* efficient silencing was measured by western blot (A) and qRT‐PCR (B) in SH‐SY5Y and NMB neuroblastoma cell lines 72 h post siRNA transfection. Data represent the mean ±SD from three independent experiments. C,D) Assessment of cell proliferation in SH‐SY5Y and NMB cell lines after silencing of *HSD17B12* (C) and in SK‐N‐BE(2) wild‐type cells and relative CRISPR/Cas9‐edited clones (clone#1, #2, and #3) (D). Cell viability measurements were performed using MTT assays at 0, 24, 48, and 72 h post siRNA transfection (C) or after seeding (D). Data shown are the mean ±SD from two independent MTT experiments, with six technical replicates for each experimental point. E,F) Representative images of trans‐well invasion assays performed in SH‐SY5Y and NMB cell lines after silencing of *HSD17B12* (E) and in SK‐N‐BE(2) wild‐type cells and relative CRISPR/Cas9‐edited clones (clone#1, #2, and #3) (F). G,H) Number of invasive cells as measured in SH‐SY5Y and NMB silenced cell lines (G) and in SK‐N‐BE(2) wild‐type cells and relative CRISPR/Cas9‐edited clones (clone#1, #2, and #3) (H). Data represent the mean ±SD from two independent experiments. * *p*‐value < 0.05; ** *p*‐value < 0.01; *** *p*‐value < 0.001. *p*‐values obtained by *t*‐test.

To further confirm the pro‐tumoral activity of *HSD17B12*, we conducted in vitro tumorigenic assays after applying RNA interference by siRNA transient transfection to diminish *HSD17B12* expression in the SH‐SY5Y and NMB neuroblastoma cells (**Figure** **4**A,B). Silencing of *HSD17B12* led to a marked decrease in cell proliferation (Figure 4C) and invasiveness (Figure 4E,G) in both neuroblastoma cell lines compared to control conditions. These results were further corroborated using an alternative gene silencing approach involving a distinct pool of siRNAs, as presented in Figure S8, Supporting Information. The consistency of results obtained with different siRNA pools reinforces the reproducibility of our data and further supports the role of *HSD17B12* in modulating cell proliferation and invasiveness.

This finding was further corroborated by comparing the aggressiveness of CRISPR/Cas9‐edited clones to the SK‐N‐BE(2) wild‐type cell line. Indeed, we observed enhanced proliferative (Figure 4D) and invasive properties (Figure 4F,H) in the CRISPR/Cas9‐edited clones relative to the SK‐N‐BE(2) wild‐type, attributable to the upregulation of *HSD17B12* following genome editing.

Collectively, these results reinforce the functional significance of the rs2863002 SNP in neuroblastoma predisposition. They highlight that this SNP modulates *HSD17B12* expression, promoting oncogenic activity in neuroblastoma by significantly enhancing tumor cell proliferation and invasiveness.

### 2.5 *HSD17B12* Silencing Alters Lipid Metabolism in Neuroblastoma Cells

**Figure 5:**
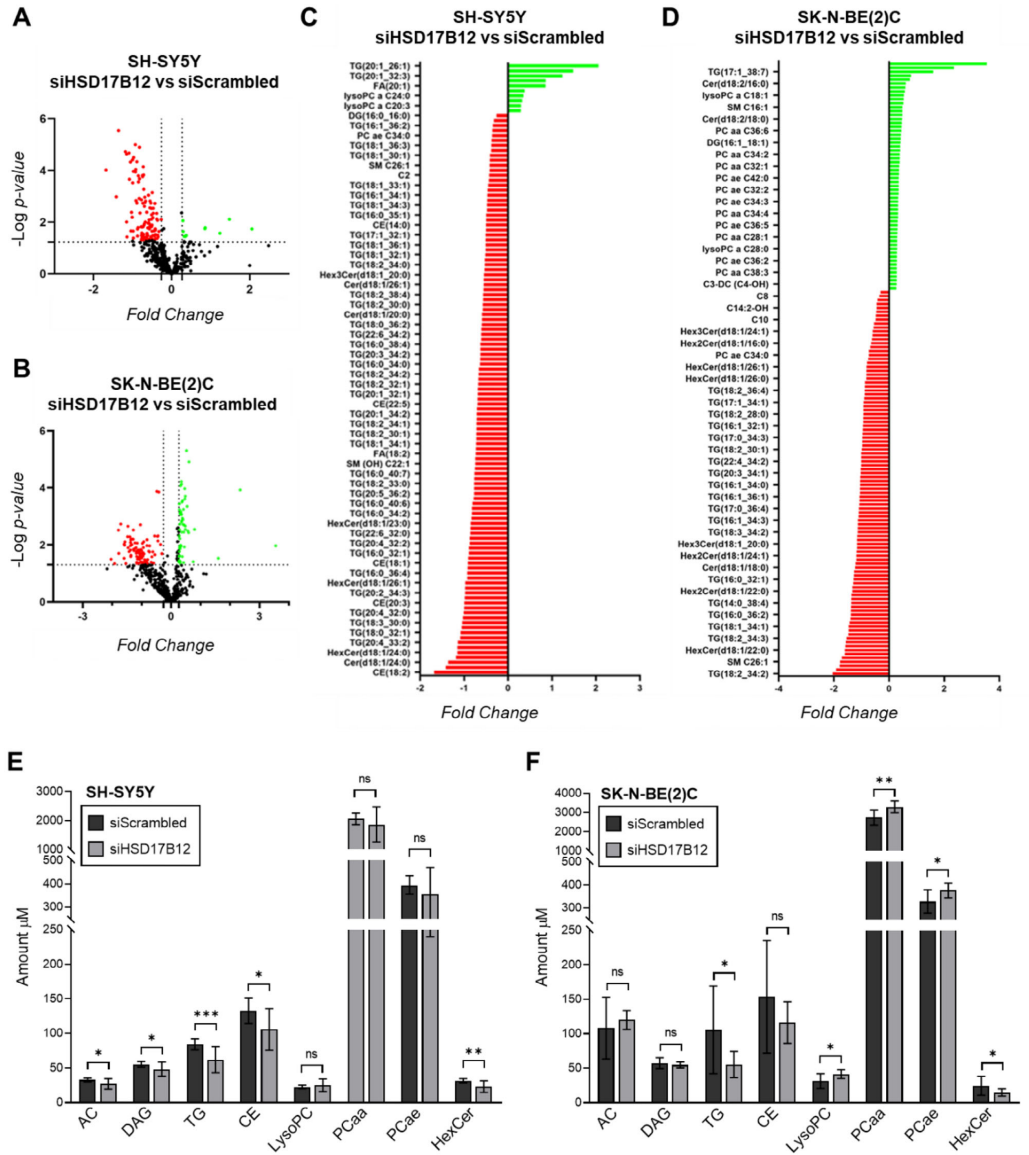
*HSD17B12* silencing affects lipid metabolism in neuroblastoma, resulting in a widespread downregulation of lipid molecules. A,B) Volcano plots of differential concentrations of 543 lipids in SH‐SY5Y (A) and SK‐N‐BE(2)C (B) cell lines after silencing of *HSD17B12* compared to their respective control. Statistically significant changed lipids were selected using *p*‐value < 0.05 (horizontal dotted line) and log2 fold change (FC) larger than ± 0.58 (vertical dotted lines). C,D) Bar plots showing the most significantly up‐ (green) and down‐regulated (red) lipids in SH‐SY5Y (C) and SK‐N‐BE(2)C (D) silenced cell lines. The differentially abundant lipids (*p* < 0.05) are plotted according to their fold change of concentration. E,F) Lipid content comparison in SH‐SY5Y (E) and SK‐N‐BE(2)C (F) neuroblastoma cells after silencing of *HSD17B12* compared to control conditions (siScrambled). Graphs represent the mean ± SD lipid amount, which indicates the sum of the metabolites' intensities within a class after normalization. ns non‐significant; * *p*‐value < 0.05; ** *p*‐value < 0.01; *** *p*‐value < 0.001. Two‐tailed unpaired *t*‐tests were performed in each lipid class to establish a statistical difference. AC: acylcarnitines; DAG: diacylglycerols; TG: triacylglycerols; CE: cholesterol esters; LysoPC: Lyso‐phosphatidylcholine; PCaa: acyl‐acyl phosphatidylcholine; PCae: alkyl‐acyl phosphatidylcholine; HexCer: glycosylceramides.

*HSD17B12* encodes for an enzyme crucial in the elongation of long‐chain fatty acids (LCFAs), a process occurring on the membranes of the endoplasmic reticulum.^[^ ^35^ ^]^ To elucidate its biological role in neuroblastoma pathogenesis, we examined *HSD17B12* function in lipid metabolism within this pediatric cancer. We performed targeted lipidomic analysis in the silenced neuroblastoma cell lines SH‐SY5Y and SK‐N‐BE(2)C, both carrying the C/C risk genotype of rs2863002, to identify lipid metabolic alterations due to *HSD17B12* silencing. We quantified 543 lipid molecules across nine major classes, including acylcarnitines (AC) (n = 40), ceramides (Cer) (n = 38), cholesterol esters (CE) (n = 22), diacylglycerols (DG) (n  =  44), fatty acids (FA) (n = 10), glycerophospholipids (LPC: Lyso‐phosphatidylcholine; PCaa: acyl‐acyl phosphatidylcholine; PCae: alkyl‐acyl phosphatidylcholine), (n  =  98), glycosylceramides (HexCer) (n  =  34), sphingolipids (SM) (n  =  15) and triacylglycerols (TG) (n  =  242) (Table S10, Supporting Information). The distribution profile of lipid species effectively distinguished between *HSD17B12*‐silenced and non‐silenced cell lines (Figure S9A,B, Supporting Information). We further analyzed the most discriminant lipid molecules for each cell line under each condition, based on their Variable Importance in Projection (VIP) scores (Figure S9C, Supporting Information). We found a predominant downregulation of various lipid molecules in both SH‐SY5Y (**Figure** **5**A,C) and SK‐N‐BE(2)C (Figure 5B,D) cell lines in response to *HSD17B12* silencing. Specifically, in the SH‐SY5Y cell line, *HSD17B12* silencing resulted in a marked decrease in the levels of acylcarnitines, diacylglycerols, triacylglycerols, cholesterol esters, and glycosylceramides (Figure 5E). No significant changes were observed in the concentrations of other lipid classes. Similarly, in the SK‐N‐BE(2)C cell line, *HSD17B12* silencing induced a significant decrease of triacylglycerols and glycosylceramides compared to controls, but there was a different trend in glycerophospholipids levels that were strongly increased (Figure 5F).

Taken together, these findings show that the silencing of *HSD17B12* has a wide impact on many cellular lipid class molecules, thus affirming an important regulatory role of *HSD17B12* in the metabolism of LCFAs within neuroblastoma.

### 2.6 rs2863002 is Implicated in Modulation of Membrane Fluidity and Lipid Droplet Abundance through Regulation of *HSD17B12*

**Figure 6:**
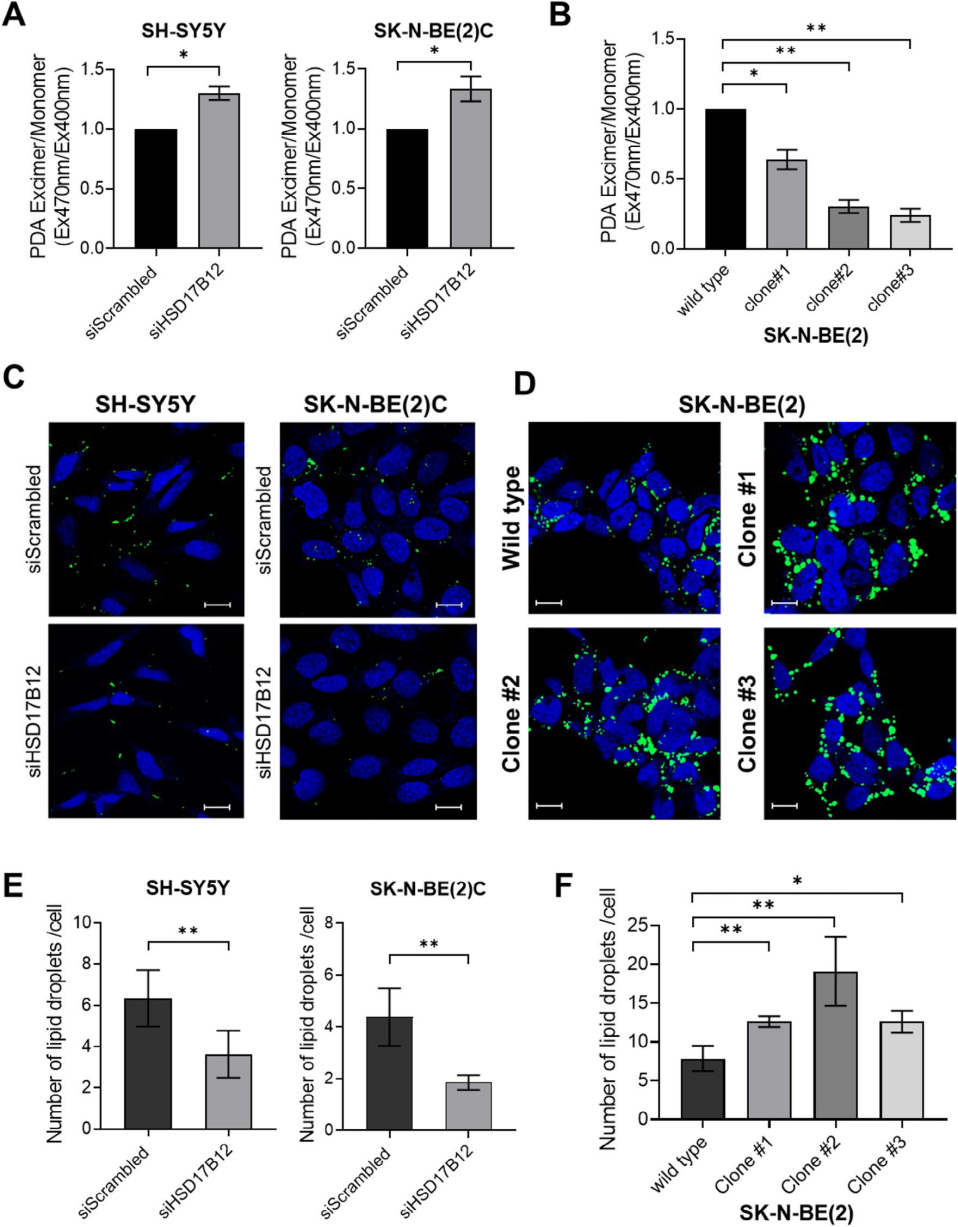
Down‐regulation of *HSD17B12* alters lipid molecules affecting the fluidity of membranes and lipid droplet properties. A,B) Membrane fluidity was assessed by measuring the ratio of pyrene‐decanoic acid (PDA) excimer to monomer fluorescence in SH‐SY5Y and SK‐N‐BE(2)C cells after silencing of *HSD17B12* (A) and in SK‐N‐BE(2) wild‐type cells and relative CRISPR/Cas9‐edited clones (clone#1, #2, and #3) (B). Fluorescence was evaluated at 400 nm for monomers and 470 nm for excimers. Data represent the mean ± SD of the measurements compared with control conditions (siScrambled) in (A) and SK‐N‐BE(2) wild type in (B) each from two independent experiments performed in duplicate. C,D) Representative confocal images of neutral lipid staining by LipidTOX Green (green) in SH‐SY5Y and SK‐N‐BE(2)C cells after silencing of *HSD17B12* (C) and in SK‐N‐BE(2) wild‐type cells and edited clones (D). Nuclei were counterstained with DRAQ5 (blue). Scale bar 20 µM. E,F) Quantification of lipid droplet number obtained through cell‐by‐cell measurements in SH‐SY5Y and SK‐N‐BE(2)C cells after silencing of *HSD17B12* (E) and in SK‐N‐BE(2) wild‐type cells and relative CRISPR/Cas9‐edited clones (F). Data represent the mean number ± SD of lipid droplets per cell; measurements have been performed on a mean number of 100 cells per experimental condition. * *p*‐value < 0.05; ** *p*‐value < 0.01. *p*‐values were calculated by *t*‐test.

To associate the changes in lipid concentrations observed following *HSD17B12* silencing to broader biophysical, chemical, and biological contexts, we performed a lipid‐ontology enrichment analysis using the LION (Lipid Ontology) online tool (http://www.lipidontology.com/).^[^ ^36^ ^]^ This analysis revealed a significant increase in lipid species functioning as membrane components and signaling molecules in both cell lines, as shown in Figure S10, Supporting Information. The endoplasmic reticulum (ER) emerged as the cellular compartment primarily affected and, particularly in SK‐N‐BE(2)C cells, there was a notable enrichment in lipid signatures indicative of high membrane fluidity, characterized by high lateral diffusion and lower transition temperatures (Figure S10A,C, Supporting Information). To empirically assess the predicted alterations in membrane fluidity, we employed a fluorescence‐based assay using the lipophilic pyrene probe, pyrene‐decanoic acid (PDA). This assay relies on the fluorescent transition of pyrene probes from dimer to excimer, which is contingent upon the spatial interaction with the cell plasma membrane; the excimer formation rate therefore serves as a proxy for membrane fluidity measurement. Our results confirmed that the silencing of *HSD17B12* using both siRNA‐based strategies, induces an increase in membrane fluidity in SH‐SY5Y and SK‐N‐BE(2)C neuroblastoma cells, in agreement with the lipid‐ontology prediction (**Figures** **6**A and S11A, Supporting Information). In contrast, CRISPR/Cas9‐edited clones with alterations of the rs2863002 SNP region exhibited decreased membrane fluidity, which can be attributed to elevated *HSD17B12* expression (Figure 6B).

In terms of the chemical and biophysical characteristics of the downregulated lipid classes, we observed a decrease in long‐chain lipid species (those with ≥ 16/18 carbon atoms) and a reduced prevalence of saturated fatty acids, which aligns with the expected consequences of inhibiting *HSD17B12* activity in the elongation of LCFAs pathway (Figure S10B,D, Supporting Information). Additionally, our lipid‐ontology enrichment analysis indicated a notable downregulation in lipid classes associated with lipid storage and energy biosynthesis in both cell lines. This reduction was predicted to predominantly impact the cellular compartment of lipid droplets (Figure S10B,D, Supporting Information).

To further prove the impact of *HSD17B12* on these lipid‐related organelles, we carried out immunofluorescence assays coupled with cell‐by‐cell measurements to assess lipid droplet size and content. These assays confirmed a significant reduction in the number and size of lipid droplets, measured by diameter and area, following the silencing of *HSD17B12* in SH‐SY5Y and SK‐N‐BE(2)C neuroblastoma cells using both siRNA‐based strategies (Figure 6C,E, Figures S11B,C and S12A–D, Supporting Information). Conversely, in CRISPR/Cas9‐edited clones with alterations of the rs2863002 region, there was a marked increase in the quantity and size of lipid droplets (Figure 6D,F, and Figure S12E,F, Supporting Information), further emphasizing the influence of the neuroblastoma risk variant rs2863002 on *HSD17B12* functionality.

Our results demonstrate that the lipid molecules mostly affected by *HSD17B12* deregulation are critical in controlling the membrane fluidity and the cellular energy compartment. By linking rs2863002 regulatory activity to biological effects on cellular metabolic pathways, these insights contribute to our understanding of the biology underlying the neuroblastoma risk locus at chr11p11.2.

## 3 Discussion

Our comprehensive post‐GWAS functional study has elucidated the biological mechanisms underlying the genetic association at the neuroblastoma susceptibility locus chr11p11.2. Through integrative genomic analysis of SNPs at the 11p11.2 locus, we focused on the variant rs2863002 T>C within *HSD17B12*, which emerged as a key regulatory element in neuroblastoma. Our validation efforts in an independent Italian cohort confirmed the association of the minor allele C of rs2863002 with increased neuroblastoma risk. Furthermore, a meta‐analysis combining data from both American‐European and Italian cohorts consolidated the genome‐wide significance of this association.

eQTL and Hi‐C analyses clarified the functional consequences of the chr11p11.2 locus, identifying *HSD17B12* as the target gene of rs2863002. We demonstrated that the C risk allele correlates with increased expression of the target gene *HSD17B12* in the adrenal gland tissue and in a set of neuroblastoma patients with the homozygous risk genotype. Genomic editing experiments using CRISPR/Cas9 system further corroborated the enhancer effect of rs2863002 on *HSD17B12* expression, underscoring the variant functional significance.

A key finding of our study is the remarkable regulatory potential of the genomic region bearing rs2863002. We discovered that this region acts as an active enhancer in multiple neuroblastoma cell lines, recapitulating the disease phenotype. Notably, we found an enrichment of binding sites for several neuroblastoma‐relevant transcription factors in this region. Of particular significance is the interaction with GATA3, a critical player in sympathetic nervous system development and neuroblastoma cell identity.^[^ ^37^ ^]^ Of note, it has been documented that GATA3 can act as either an activator or repressor of its target genes.^[^ ^38^ ^]^ Our results suggest that the C risk allele of rs2863002 alters a GATA3 binding motif, reducing its binding affinity and leading to increased *HSD17B12* expression. This finding provides a mechanistic link between genetic variation and gene regulation in neuroblastoma development.

*HSD17B12* has been established as a gene of oncological importance across diverse tumor types through numerous GWASs, with associations observed in cutaneous melanoma, prostate, colorectal, and oral cancer,^[^ ^39^, ^40^, ^41^, ^42^ ^]^ but its functional role in neuroblastoma was unexplored. Furthermore, the literature consistently reports that silencing *HSD17B12* can markedly decrease the proliferation and invasion of ovarian and breast cancer cells.^[^ ^30^, ^31^, ^32^ ^]^ A noteworthy addition to this body of evidence is a recent study associating the upregulation of *HSD17B12* with poor prognosis and lower event‐free survival rates in neuroblastoma.^[^ ^43^ ^]^ Indeed, in agreement with these very recent findings, we found that higher *HSD17B12* expression is associated with negative prognostic indicators in neuroblastoma. Additionally, we demonstrated that suppression of *HSD17B12* in neuroblastoma cell models significantly reduces tumor cell growth and invasiveness. This effect is underscored by our observations of enhanced malignancy in isogenic cell lines with genetic variations in the rs2863002 locus, which are characterized by the upregulation of *HSD17B12*. Collectively, these results robustly sustain the contribution of the genetic variant rs2863002 and its regulatory effect on *HSD17B12* to the oncogenic process in neuroblastoma.

Our investigation into the role of *HSD17B12* in lipid metabolism provides another layer of significance to this study. HSD17B12 was initially characterized as a pivotal enzyme in the steroid metabolism pathway but its role in cancer has been linked to lipid metabolism.^[^ ^30^, ^44^ ^]^ This gene encodes a reductase responsible for catalyzing the second of four reactions of the fatty acids elongation pathway which produces long‐chain (LCFAs) and very long‐chain fatty acids (VLCFAs), containing more than 18 and 22 carbon atoms, respectively.^[^ ^35^ ^]^ The reprogramming of lipid metabolism is a recognized hallmark of cancer cell biology, essential for supporting the growth, division, and survival of malignant cells.^[^ ^45^, ^46^ ^]^ Lipids are not only structural components of cellular membranes but also serve as signaling molecules and energy sources that support rapid cell proliferation.^[^ ^47^ ^]^ In neuroblastoma, increased lipid biosynthesis and desaturation have been associated with *MYCN* amplification and tumor aggressiveness.^[^ ^46^, ^47^ ^]^ Moreover, changes in the lipidome profile has been linked with mechanisms such as oxidative stress responses and susceptibility to ferroptosis—a regulated form of lipid peroxidation‐dependent cell death.^[^ ^48^, ^49^, ^50^ ^]^ These mechanisms are particularly relevant in neuroblastoma, where lipid homeostasis appears to intersect with both survival and differentiation pathways.

Our investigation of lipid metabolism following the silencing of *HSD17B12* or genome editing on the rs2863002 SNP revealed a significant impact on the lipid profiles and downregulation of key lipid molecules in these in vitro neuroblastoma cellular models. Our comprehensive lipidomic analysis, spanning a wide spectrum of lipid species, highlighted that the resultant alterations in lipid metabolism influence structural components of cellular membranes and lipid species linked to energy storage and production. These changes directly impact membrane fluidity and lipid droplet properties, which have been demonstrated to affect tumor cell behavior and pathophysiology.^[^ ^51^, ^52^, ^53^ ^]^

Our results confirm that *HSD17B12* contributes to oncogenesis by bolstering the synthesis of fatty acids in human cancers, as highlighted by Nagasaki et al. and Szajnik et al.^[^ ^31^, ^32^ ^]^ Moreover, in agreement with Mohamed et al.,^[^ ^54^ ^]^ we demonstrated that *HSD17B12* modulation significantly affects the number and dimension of lipid droplets, which are key indicators of cellular energy stores and recognized hallmarks of cancer cell aggressiveness.^[^ ^52^ ^]^

Importantly, we uncovered a previously unrecognized link between genetic susceptibility and lipid metabolism in neuroblastoma. By a combination of genome editing experiments and functional validation of the lipidomic results, we demonstrated that the altered lipid metabolism in neuroblastoma cells was likely mediated by the risk SNP rs2863002 via modulation of *HSD17B12* expression. This discovery provides deeper insight into the biological mechanisms at the 11p11.2 locus that are associated with an increased susceptibility to neuroblastoma.

The implications of this study extend into the clinical realm, highlighting novel therapeutic targets for neuroblastoma treatment. The identification of *HSD17B12* as a novel neuroblastoma susceptibility gene involved in lipid metabolism suggests it could serve as a biomarker for risk stratification and a potential therapeutic target. Indeed, lipid metabolism has been gaining interest in the neuroblastoma field lately as it offers novel and promising therapeutic strategies.^[^ ^55^ ^]^ For instance, inhibiting β‐oxidation with Etomoxir diminished in vivo tumor growth in *MYCN*‐amplified neuroblastoma cells,^[^ ^56^ ^]^ while small‐molecule inhibitors of *de novo* fatty acid synthesis reduced neuroblastoma growth both in vitro and in vivo.^[^ ^57^ ^]^ Of note, an ongoing clinical trial (ClinicalTrials.gov identifier: NCT04565717) is testing the use of *HSD17B13* inhibitors to treat liver steatosis. With some similarities to *HSD17B12*, *HSD17B13* was recently shown to be a lipid droplet‐associated protein involved in lipid homeostasis in the liver.^[^ ^58^, ^59^ ^]^ Future research should explore the clinical utility of *HSD17B12* expression levels or the rs2863002 variant genotype in personalizing neuroblastoma treatment. Moreover, the link between genetic risk and lipid metabolism unveiled in this study may have broader implications for cancer biology, potentially applicable to other pediatric and adult cancers.

While our study provides strong evidence that molecular mechanisms at the 11p11.2 locus significantly contribute to neuroblastoma pathogenesis, several limitations should be acknowledged. First, we focused exclusively on a single non‐coding variant, rs2863002, in *HSD17B12*, without examining other potential functional regulatory SNPs at the same locus. In this study, we provide a list of additional candidate regulatory SNPs that may influence *HSD17B12* or other target genes, potentially contributing to neuroblastoma development. Future genomic investigations are necessary to dissect the functional role of these variants or target genes implicated in the disease. Second, the allele frequency of rs2863002 and its impact on neuroblastoma risk may vary among different populations. We have demonstrated the genetic association of the variant in European Americans and Italians. Further studies are needed to determine whether these findings are consistent across diverse ethnic groups, such as African, Hispanic, or Asian individuals. Third, although the lipidomic analysis was comprehensive, it may not capture all lipid species, especially those present at low abundance or transient intermediates. Advanced techniques or untargeted lipidomic analyses could reveal additional lipid alterations. Finally, although our in vitro findings and lipidomic data are statistically robust, we acknowledge that the observed changes are modest in magnitude. Validation in preclinical in vivo models, such as xenografts, will be an important next step to confirm the biological impact of these alterations on neuroblastoma growth.

In conclusion, our study not only provides insights into the genetic basis of neuroblastoma susceptibility but also bridges the gap between genetic risk factors and cellular metabolism in cancer development. The original approach we employed, combining genetic association studies with functional genomics and lipidomics, has unveiled a complex regulatory mechanism involving the rs2863002 variant, *HSD17B12* expression, and lipid metabolism in neuroblastoma pathogenesis. These findings contribute significantly to the growing body of knowledge on cancer genomics and metabolism, paving the way for innovative approaches in neuroblastoma management and potentially in other cancer types.

## 4 Experimental Section

### Prioritization of Causal Variants at 11p11.2 Risk Locus

This work first selected the variants in linkage disequilibrium with the lead SNP rs10742682 (r^2^>0.8) (n = 42) at the 11p11.2 locus in the European‐American population using LDlink (analysistools.cancer.gov/ LDlink). To prioritize potential functional variants, this work annotated these SNPs with multiple sources of in silico functional annotations from public databases and from newly in‐house generated data. To identify SNPs mapping inside open chromatin regions this work generated the transposase‐accessible chromatin using sequencing (ATAC–seq) profiles of the neuroblastoma cell lines CHP‐134, CHP‐212, GI‐ME‐N, IMR‐32, LAN‐2, SH‐EP, SH‐SY5Y, SK‐N‐BE(2) and SK‐N‐BE(2)C. Moreover, public ATAC‐seq reads from the neuroblastoma cell lines COG‐N‐440, COG‐N‐453, COG‐N‐415, LAN‐5, NB‐1, NB‐1643, NB‐69, NBL‐S, SK‐N‐FI, SK‐N‐SH (GSE138315), SH‐EP‐21N (GSE80152), KELLY (GSE138315, GSE80152, GSE94824), NGP (GSE138315, GSE80152), SK‐N‐AS (GSE138315) and SK‐N‐BE(2)C (GSE138315, GSE80152, GSE94824) were downloaded from the NCBI Gene Expression Omnibus (GEO) public database and used as processed files. In total, this work analyzed 44 ATAC‐seq experiments on 22 different neuroblastoma cell lines. Similarly, the DNase‐I hypersensitivity level (DHS) signal intensity files from the SK‐N‐BE(2)C (GSE29692) and SK‐N‐SH (GSE32970) were downloaded from the GEO database. Furthermore, to prioritize SNPs mapping in active enhancer elements, this work analyzed H3K27ac chromatin immunoprecipitation sequencing (ChIPseq) data to study the epigenomic profiles of COG‐N‐415, KELLY, LA‐N‐5, NB‐1643, NBL‐S, NGP (GSE138315), CHP‐212, CLB‐BER‐Lud, CLB‐CAR, CLB‐GA, CLB‐MA, CLB‐PE, GI‐CA‐N, GI‐ME‐N, IMR‐32, LA‐N‐1, N206, NB‐EBc1, SH‐EP, SJNB‐1, SJNB‐12, SJNB‐6, SJNB‐8, SK‐N‐DZ, SK‐N‐SH, TR14 (GSE90683), 691‐ADN, 700‐ADN, 700‐MES, 711‐ADN, 717‐MES, 753‐ADN, SH‐EP (GSE90805), NB‐69, SK‐N‐AS, SK‐N‐FI, SK‐N‐BE(2)‐C (GSE138315, GSE90683) and SH‐SY5Y (GSE90683, GSE90805), for a total of 43 H3K27ac ChIP‐seq experiments from 37 different neuroblastoma cell lines. Functionally informed fine mapping was carried out with PolyFun, in combination with the SuSiE algorithm.^[^ ^33^, ^60^ ^]^ PolyFun estimates SNP‐specific prior causal probabilities by leveraging functional annotations from UKBB including coding, conserved, regulatory and LD‐related annotations, and LD reference panels from 1000 Genomes (EUR). Subsequently, SuSiE was used to compute posterior inclusion probabilities (PIPs) and derive 95% credible sets for each locus, thereby improving causal inference by integrating biological information with statistical fine‐mapping.^[^ ^60^ ^]^

### Assay for Transposase‐Accessible Chromatin Using Sequencing in Neuroblastoma Cell Lines

ATAC‐seq experiments were performed by AZENTA Life Sciences. According to GENEWIZ‐protocol, CHP‐134, CHP‐212, GI‐ME‐N, IMR‐32, LAN‐2, SHEP‐2, SH‐SY5Y, SK‐N‐BE(2) and SK‐N‐BE(2)‐C frozen cell suspensions were prepared. A total amount of 1500000 cells were cryopreserved in cell culture media supplemented with 10% DMSO and sent for sequencing.

Public and in‐house ATAC‐seq data were downloaded and re‐analyzed as follows. Fastq files were processed using Nextflow and nfcore‐core ATAC‐seq v1.2.2 pipelines.^[^ ^61^ ^]^ In brief, read adapters were trimmed using Trim Galore; alignment was performed using BWA; duplicates were removed, and peaks were called using MACS2 in narrow peak mode.^[^ ^62^ ^]^ Peaks with FDR ≤ 0.01 were retained.

### Neuroblastoma Replication in a Cohort of Italian Patients

Genomic DNA of neuroblastoma patients and healthy controls was extracted from peripheral blood using the QIAamp DNA mini kit (Qiagen). DNA concentration and purity were evaluated using the Qubit 4.0 Fluorometer from Thermo Fisher. The rs2863002 SNP was typed by TaqMan SNP Genotyping Assay (Applied Biosystems, Thermo Fisher Scientific, Waltham, MA, USA) in an Italian cohort of 607 neuroblastoma cases and 2032 controls. Hardy‐Weinberg equilibrium was evaluated using the goodness‐of‐fit chi‐square test in control and case subjects. Statistical significance was set at *p*‐value < 0.05. Logistic regression, with age and sex as covariates, was used to evaluate differences in the distributions of allele frequencies between patients and controls. Odds ratios (ORs) and 95% confidence intervals (CIs) were calculated to assess the relative disease risk conferred by the specific alleles.

Results from the Italian cohort and the published neuroblastoma GWAS summary statistics were combined using an inverse variance‐weighted meta‐analysis according to the formulas described in Willer et al.^[^ ^63^ ^]^ Notably, to combine the results from the validation cohort with data from the European‐American population in the common meta‐analysis, these results have been re‐calculated by logistic regression, using age and sex as covariates. This study was approved by the Ethics Committee of the Federico II University of Naples.

### Identification of Transcription Factor Motifs Altered by rs2863002

To identify significantly enriched TF motifs covering the rs2863002 genomic position, this work defined a 50 bp region centered at this SNP using the GenomicRanges R package (https://bioconductor.org/packages/release/bioc/html/GenomicRanges.html). Next, this work applied a motif enrichment analysis using the PWMEnrich R package (https://www.bioconductor.org/packages/release/bioc/html/PWMEnrich.html). Only binding motifs with a *p*‐value ≤ 0.05 were considered significantly enriched. Subsequently, this work leveraged the FABIAN tool to assess which TF binding motifs were gained or disrupted by the two different alleles of rs2863002. FABIAN is a web‐based application that uses transcription factor flexible models (TFFMs) and position weight matrices (PWMs) to infer the extent to which DNA variants affect TF motif affinity.^[^ ^64^ ^]^ The FABIAN output is a score per Transcription Factor binding site (TFBS) per variant, ranging from 1 to −1, with 0 reflecting any variant effects on TF binding, positive scores indicating an increased binding affinity, and negative scores indicating decreased binding affinity.

To confidently select motifs affected by rs2863002, this work chose those found significantly enriched in PWMEnrich analysis, characterized by a FABIAN score ≥ +0.1 or ≤ – 0.1 and interacting with TF whose binding was confirmed by ChIP‐seq experiments in neuroblastoma cell lines.

### Analysis of Public ChIP‐seq Data and In‐House ChIP‐seq Experiments

To assess transcription factor binding at rs2863002 genomic location, this work downloaded and analyzed ChIP‐seq experiments for TFs such as GATA3, MYCN, ASCL1, LMO1, HAND2, ISL1, PHOX2B and TBX2 in the neuroblastoma cell lines COG‐N‐415, LA‐N‐5, NB‐1643, NB‐69, KELLY, NGP, SH‐EP‐21N, SH‐SY5Y and SK‐N‐BE(2)C deposited in NCBI Gene Expression Omnibus (GEO) with accession numbers GSE138315, GSE120074, GSE80151, GSE94782, GSE94824, GSE65664, GSE169616. In total, this work analyzed 44 ChIP‐seq experiments to assess TFs binding in 10 neuroblastoma cell lines. Fastq files of public ChIP‐seq experiments were processed using Nextflow together with the nf‐core ChIP‐seq v1.2.2 pipelines.^[^ ^61^ ^]^ In brief, read adapters were trimmed using Trim Galore and alignment was performed using BWA; duplicates were removed, and peaks were called using MACS2 in narrow peak mode.^[^ ^62^ ^]^ Peaks with FDR ≤ 0.01 were retained. Quality control parameters, such as the fraction of reads in peaks (FRiP), normalized strand cross‐correlation coefficient (NSC), and relative strand cross‐correlation coefficient (RSC), were determined according to the Encyclopedia of DNA Elements (ENCODE) guidelines.^[^ ^65^ ^]^ TF motif enrichment analysis was performed using Homer v4.11 findMotifsGenome.pl tool^[^ ^66^ ^]^ on each set of ChIP‐seq peaks specific to each neuroblastoma cell line. Results were filtered for highly enriched (*p* < 1 × 10^−10^ and Target sequences >5%) TF binding motifs.

### Chromatin Immunoprecipitation Sequencing Experiments in Neuroblastoma Cell Lines

We performed new in‐house ChIP‐seq experiments to analyze the binding profile of the TFs GATA3 in the SH‐SY5Y and SK‐N‐BE(2)C cell lines, MYCN in the SK‐N‐BE(2)C cell line, JUN and FOSL2 in the GI‐ME‐N and SH‐EP cell lines, and FOSL1 and PRRX1 in the SH‐EP cell line. ChIP experiments were performed by using iDeal ChIP‐seq kit for Transcription Factors (C01010055, Diagenode) according to manufacturer instructions. Briefly, 10–12 million cells per IP reaction were fixed by formaldehyde at a final concentration of 11% to obtain DNA‐protein cross‐linking. After cell lysis, fragmentation of the cross‐linked chromatin was performed using Diagenode Bioruptor Plus (Diagenode) by 30 cycles of sonication. Protein A magnetic beads were used to isolate the protein‐DNA complexes of interest. Chromatin immunoprecipitation was obtained by using GATA3 (ab199428, Abcam), MYCN (ab227822, Abcam), JUN (ab32137, Abcam), FOSL1 (sc‐28310 C‐12X, Santa Cruz Biotechnology), FOSL2 (#19 967, Cell Signaling) and PRRX1 (SAB1412737, Sigma) ChIP‐grade antibodies and a normal Rabbit IgG antibody (supplied by the kit) as a negative control. After overnight incubation on a rotating platform at 4 °C, samples were purified to remove the chromatin proteins and to prepare the DNA for the following detection steps. After DNA quantification, samples were prepared to a final concentration of ≥2 ng µL^−1^ and sent to Novogene for sequencing with the Illumina sequencing platform.

Fastq files from the in‐house ChIP‐seq experiments were processed using the same pipelines described above for the analysis of public ChIP‐seq data experiments.

### Chromatin Immunoprecipitation qPCR

This work assessed the binding affinity of the TFs GATA3 and MYCN for the two different alleles of rs2863002 by performing ChIP qPCR experiments in SH‐SY5Y (C/C genotype) and NMB (T/T genotype) cell lines, using a GATA3 ChIP‐grade antibody (ab199428; Abcam) and a MYCN ChIP‐grade antibody (ab227822; Abcam). To validate GATA3‐ChIP reactions, this work measured the chromatin fold enrichment in the specific region containing rs2863002 and in a positive and negative control region: as a positive binding control, this work selected an intergenic region (>chr2:15982438–15982518) predicted to bind GATA3 with a high H3K27ac peak in neuroblastoma cell lines Chip‐seq experiments, while a genetic region proximal to *CDH10* gene (>chr5:24682868–24682979) was used as negative control.^[^ ^67^ ^]^ To validate MYCN‐ChIP reactions, this work measured the chromatin fold enrichment in the specific region containing rs2863002 and in a positive and negative control region: as a positive binding control, this work selected a region (>chr5:170814842–170815848) predicted to bind MYCN in neuroblastoma cell lines ChIP‐seq experiments, while a genetic region with no significant TF motif enrichment (>chr2:80521594–80524257) was used as negative control.^[^ ^67^ ^]^ The primer sequences are listed in Table S11, Supporting Information.

ChIP reactions were performed using iDeal ChIP‐seq kit for Transcription Factors (C01010055, Diagenode) according to manufacturer instructions, as described above for ChIP‐seq experiments. In brief, cross‐linking of DNA‐bound proteins was performed by fixing ≈10–12 million SH‐SY5Y and NMB cells with a solution of formaldehyde to an 11% final concentration in growth media. After cell lysis, sonication of cells was performed using a Diagenode Bioruptor sonicator to shear the chromatin to 200–800 bp fragments. Gel electrophoresis analysis was used to empirically confirm the efficiency of chromatin shearing. Chromatin fragments were immunoprecipitated using A/G‐conjugated magnetic beads together with GATA3 primary antibody (ab199428 Abcam), MYCN primary antibody (ab227822 Abcam), and normal Rabbit IgG (supplied by the kit) as a negative control. Samples were incubated overnight on a rotating platform at 4 °C. After sequential higher stringency washes, antibody/chromatin complexes were eluted, and protein–DNA cross‐links were reversed by overnight incubation at 67 °C with shaking. DNA was purified to remove the chromatin proteins and to prepare the DNA for the following detection steps. Real‐time quantitative PCR (qPCR) was performed following the manual's guidelines. Experiments were performed in triplicate for each cell line and the statistical significance of the results was assessed using unpaired student's *t*‐test.

### Cell Cultures

The human SH‐SY5Y (DSMZ, ACC 209) and NMB (DSMZ, ACC 657) cell lines were grown in Dulbecco's Modified Eagle Medium (DMEM, Sigma); the human SK‐N‐BE(2)C (ATCC, CRL‐2268) and LAN‐2 (ECACC, 0 604 1202) were grown in a 1:1 mixture of Minimum essential Medium Eagle (EMEM, Lonza) and Nutrient Mixture F12 (Sigma); the SK‐N‐BE(2) (ATCC, CRL‐2271) cell line was grown in a 1:1 mixture of Dulbecco's Modified Eagle Medium (DMEM, Sigma) and Nutrient Mixture F12 (Sigma); the human CHP‐134 (ECACC, 0 612 2002), GI‐ME‐N (DSMZ, ACC654) and SH‐EP (ATCC, CRL‐2269) cell lines were grown in RPMI‐1640 Medium (Sigma); the human IMR32 (ATCC, CRL‐127) cell line was grown in Minimum Essential Medium Eagle (MEM, Sigma); the human CHP‐212 (ATCC, CRL‐2273) cell line was grown in a mixture of 1:1 Minimum Essential Medium Eagle (MEM, Sigma) and Nutrient Mixture F12 (Sigma). In all cases, the medium was supplemented with 10% heat‐inactivated FBS (Sigma), 1vmmol/L L‐glutamine, penicillin (100 U mL^−1^), and streptomycin (100 mg mL^−1^; Invitrogen). Cells were cultured at 37 °C, 5% CO_2_ in a humidified atmosphere and only early‐passage cells were employed. All cell lines were authenticated by STR profiling and tested as mycoplasma‐free.

### CRISPR/Cas9 Genome Editing

To explore the biological effects of rs2863002, this work used the CRISPR/Cas9 system to introduce random INDELs around the SNP position (hg19/chr11:43 714 768) as previously described.^[^ ^34^ ^]^ In brief, a guide RNA (gRNA, 5′‐GATTGATTAAAAGCAACGAT‐3′) was designed using the CRISPOR Tool (http://crispor.tefor.net/) and cloned into a pSpCas9(BB)‐2A‐GFP (PX458) vector expressing Cas9 (Addgene). The obtained construct was transfected into SK‐N‐BE(2) cells using Transfectin Lipid Reagent (Bio‐Rad) and, 48 h post‐transfection, GFP‐positive cells were FACS‐sorted at single cell level into 96‐well plates. DNA was extracted by using the Wizard Genomic DNA Purification Kit (Promega) and successful genome editing was established by PCR and Sanger sequencing. PCR was performed with the KAPA HiFi HotStart PCR Kit (Roche) following the manufacturer's instructions, using the following primers (M13 sequence is highlighted in lower case):

Forward 5′‐tgtaaaacgacggccagtACACAAGTTTAGTCAGCCATTTTGT‐3′

Reverse 5′‐caggaaacagctatgaccGTTCTGCCAGCATCCTCATC‐3′

Electropherograms were analyzed using the web tool Synthego ICE (ice.synthego.com) to calculate the percentage of editing efficiency.

### Dual‐Luciferase Reporter Assay

The genetic region spanning from about 500Kb upstream to 500Kb downstream rs2863002 variant was cloned in a pGL3‐promoter vector (Promega, Madison USA) containing the Firefly luciferase reporter gene. PCR primers containing recognition sites for NheI (forward) and XhoI (reverse) were designed to amplify 1.111 bp from the genomic DNA of cell lines homozygous for the two genotypes of rs2863002, namely SH‐SY5Y (C/C genotype) and NMB (T/T genotype). The primers used are (restriction sites highlighted in bold):

Forward 5′‐ **AAAACTAGCTAGCTAG**AGAGTGTGTTGACCAAAATGTAGT‐ 3′

Reverse 5′‐ **CCGCTCGAGCGG**TTCCTACTGGAAGTTAGCTTGG‐ 3′

PCR products were double digested using NheI and XhoI restriction enzymes (Biolabs) and cloned inside linearized pGL3‐Promoter vector plasmids (Promega, Madison USA). The sequences of the inserts from the obtained constructs were confirmed by direct Sanger sequencing before transient transfection. The neuroblastoma cell lines SH‐SY5Y and SH‐EP, and the non‐ neuroblastoma cells HEK293 were co‐transfected using X‐tremeGENE (Roche) with pGL3‐rs2863002‐C or pGL3‐rs2863002‐T constructs and pRL‐TK Renilla Luciferase Control Vector (Promega, Madison USA) as a normalizing control. Cells were harvested 24 h post‐transfection, lysed, and analyzed for luciferase activity using the Dual‐Luciferase Reporter Assay System (Promega, Madison USA) on a TD20/20 Luminometer (Turner Designs). Relative luciferase activity results were obtained by normalization of the Firefly luciferase activity to Renilla luciferase activity. Data represent the means ± SD of three independent transfections. Statistical significance of the results was assessed using unpaired student's *t*‐test.

### Expression Quantitative Trait Loci Analysis

To identify genes regulated by the variant of interest, this work analyzed expression quantitative loci traits (eQTLs) data from the adrenal gland tissue (N = 295) using the public GTEx database v8 (https://gtexportal.org/home).

Moreover, this work used WGS and RNA‐seq data of neuroblastoma patients from the Target project (accession no.: phs000218.v21.p7; project ID: #14 831)^[^ ^9^ ^]^ to analyze gene expression levels in comparison with rs2863002 genotypes. RNA‐seq data were available for 161 samples in the form of processed fragments per kilobase of exon model per million reads mapped (FPKM), but we included in the analysis only samples for which both WGS and gene expression data were available (N = 89). Wilcoxon‐Mann‐Whitney test was used to compare the differences in the mRNA expression levels in the groups with different rs2863002 genotypes.

### In‐House Hi‐C Analysis

In‐house Hi‐C data of the SK‐N‐BE(2) cell line were processed as reported previously.^[^ ^68^ ^]^ After sequencing using an Illumina HiSeq platform, paired‐end reads of 150 base pairs (bp) were aligned to the reference genome (build hg19/GRCH37) using Bowtie2. HiCExplorer (v3.7) was used to (1) build the interaction matrix at a resolution of 10 Kb (bin size = 10 Kb), (2) normalize the observed read counts, (3) determine topologically associating domains (TADs, self‐interacting genome regions) and their boundaries, and (4) plot the results. Subsequently, starting from the genomic location of rs2863002 (chr11:43 714 768), this work extended the region of interest of 0.4 Mb up‐ and downstream and calculated the statistical significance of the interactions between bins with FitHiC (v2.0.8). *p*‐values were corrected for multiple tests using the Benjamini‐Hochberg method (false discovery rate, FDR), and the cutoff was set at 5%. Finally, this work annotated these bins using the R‐Bioconductor package ChIPseeker (v 1.32.0) to map the genomic bins to gene coordinates.

### In Silico Hi‐C Data Analyses

This work examined public Hi‐C sequencing data obtained on the neuroblastoma cell line SK‐N‐DZ and hosted at the 3D‐genome Interaction Viewer (3DIV), to analyze chromatin interactors of rs2863002. 3DIV was run using distance‐normalized interaction frequency ≥ 2 to define significant enhancer‐promoter interactions.

### RNA Interference by siRNA Transfection

To perform *HSD17B12* silencing, cells were seeded in 6‐well plates at a density of 2,5/3 × 10^5^ cells per well. As a first silencing strategy, neuroblastoma cells were transfected with a combination of three unique 27mer siRNA duplexes (pooled siRNA A‐B‐C) targeting *HSD17B12* (SR324050, Origene), or SR30004‐Universal Scrambled Negative Control siRNA (Scrambled) duplex (Origene). As a second gene silencing strategy, neuroblastoma cells were transfected with a distinct pool of four siRNAs (ON‐TARGETplus Human HSD17B12 SMARTpool siRNAs, L‐008474‐02, Horizon Discovery) targeting different non‐overlapping regions of the gene. As a negative control, cells were transfected with the ON‐TARGETplus Non‐Targeting Control siRNA #1 (D‐001810‐01, Horizon Discovery). Transfections were performed using the X‐tremeGENE siRNA Transfection Reagent (Sigma‐Aldrich), following the manufacturer's protocol. Cells were harvested 72 h post‐transfection and *HSD17B12* efficient silencing was assessed by Western blot and Real‐time quantitative PCR (qPCR).

### Western Blotting

HSD17B12, GATA3, and β‐Actin protein levels were analyzed by Western blotting. Cell pellets were re‐suspended and lysed in RIPA buffer (10 mM Tris‐HCl pH 8.0, 1 mM EDTA, 0.5 mM EGTA 1%, Triton X‐100, 0.1% sodium deoxycholate, 0.1% SDS and 140 mM NaCl) in presence of a protease inhibitors cocktail (Roche). Total protein extract concentrations were determined by Bradford assays (Bio‐Rad). Samples were loaded and separated using 10% polyacrylamide gels, then transferred onto polyvinylidene difluoride (PVDF) membranes (Bio‐Rad). After 1 h blocking with 5% non‐fat dried milk (Applichem) in phosphate‐buffered saline (PBS) with 0.1% Tween (PBS‐T) and washes in PBS‐T, membranes were probed overnight at 4 °C with rabbit anti‐KAR (synonym of *HSD17B12*) antibody (1:5000 dilution, ab236990 Abcam) and recombinant rabbit anti‐GATA3 antibody (1:5000 dilution, ab199428 Abcam). A mouse anti‐β‐actin antibody (1:10 000 dilution, A2228 Sigma) was used as loading control. After membrane incubation with horseradish‐peroxidase (HRP)‐conjugated anti‐rabbit and anti‐mouse IgG antibody (1:4000 dilution, ImmunoReagents), positive bands were visualized using the ECL kit Super Signal West Pico Chemiluminescent Substrate (Pierce) through expositions to autoradiograph (AR) X‐ray films. Digital images were analyzed using ImageJ software.

### Real‐Time Quantitative PCR

The mRNA expression levels of *HSD17B12*, *GATA3*, and *β‐Actin* were analyzed using Real‐time quantitative PCR. Total RNA samples were extracted using TRIzol LS Reagent (Invitrogen) and cDNA retro‐transcription was performed using the SensiFAST cDNA Synthesis Kit (Bioline) according to the manufacturer protocols. Gene‐specific primers were designed by using PRIMER EXPRESS software (Applied Biosystems) and their sequence is reported in Table S11, Supporting Information.

Real‐time quantitative PCR (qPCR) reactions were performed using SYBR Green PCR Master Mix (Applied Biosystems) in the 7900HT Fast Real‐Time PCR System (Applied Biosystems). Experiments were carried out in triplicate for each data point. Relative gene expression was obtained using the 2−ΔCT method, where the ΔCT was calculated using the differences in the mean CT between the selected gene and the internal control (β‐actin).

### Cell Viability Assay

SH‐SY5Y and NMB cells were seeded in six replicates into 96‐well plates at a density of 1 × 10^4^ cells per well. After 12 h of incubation to allow cell attachment, siRNA transfection was performed as described before for each well. Cell viability was measured at 0 h, 24 h, 48 h, and 72 h post‐transfection by evaluating metabolic conversion made by viable cells of the 3‐(4,5‐dimethylthiazol‐2L)‐2,5‐diphenyltetrazolium bromide (MTT) dye, according to the manufacturer protocol (Promega). At each selected time point, MTT assay stock solution was added to the cells and left in incubation for 3–5 h. The insoluble salts produced by the cells during the MTT conversion were dissolved by the addition of dimethyl‐sulfoxide (DMSO) and absorbance was determined spectrophotometrically at 570 nm using a reference wavelength of 620 nm, on an EnSpire Multimode Plate Reader (PerkinElmer). The experiments have been repeated twice using six‐ replicates per condition. The statistical significance of the results was assessed using an unpaired student's *t‐*test.

### Invasion Assay

To perform invasion assays, this work used specific trans‐well chambers (8 µm pore size PET, Corning, NY, USA) pre‐coated with Collagen‐I (Sigma‐Aldrich). Cells were first transfected with *HSD17B12*·and control siRNAs, then seeded in FBS‐deprived growth media on the upper compartment of the trans‐well chambers. Complete growth media supplemented with 10% FBS was added to the lower compartment of the trans‐well chambers thus creating a crescent FBS gradient between the upper and lower compartments. Cells were incubated for 24 h at 37 °C with 5% CO_2_ to allow the migration from the lower to the higher FBS concentration medium side. Migrated cells were stained with Hematoxylin & Eosin (Sigma‐Aldrich) and invading cells were counted using the Leica Application Suite/AF software and DMI4000B microscope (Leica Microsystem). Images were acquired using 5× to 20× magnification. The experiments were performed in duplicate and five experimental points were analyzed for each condition. The statistical significance of the results was assessed using an unpaired student's *t*‐test.

### LC‐MS/MS Targeted Lipidomic Approach

Lipid species fluctuations were analyzed by using a targeted Lipidomic approach combining liquid chromatography with tandem mass spectrometry (LC‐MS/MS) as previously described.^[^ ^69^ ^]^ *HSD17B12* expression was silenced in SH‐SY5Y and SK‐N‐BE(2)C cells by siRNA transfection as described before and four biological replicates were used for each condition in the present analysis. Cellular pellets were homogenized in cold methanol and centrifuged to separate proteins and cell debris from the supernatant. According to Mxp Quant 500 protocols (Biocrates Life Sciences Innsbruck, Austria), the Lipidomic mass spectrometry‐based platform was set to measure the concentration of more than 500 lipids. Three technical replicates were carried out for each sample. Lipids were extracted with 5 mM ammonium acetate in methanol and tandem mass spectrometry analysis was performed setting the multiple reaction monitoring (MRM) to identify and quantify species belonging to different lipid classes. Direct flow injection analysis (FIA) was carried out using Agilent 1260 Infinity II HPLC (High‐performance liquid chromatography), online with a Triple Quad 5500 + System QTrap‐Ready (AB Sciex). Data were acquired using Analyst software (version 1.7.1 Ab Sciex) and analyzed using MetIDQ Oxygen 2976 (Biocrates Life Sciences Innsbruck, Austria). The identified lipids were annotated according to LIPID MAP database (https://www.lipidmaps.org). The concentrations of more than 500 lipids were measured and statistically analyzed by univariate method. The significant difference was established by performing an unpaired *t*‐test with Welch's correction in normally distributed datasets or Mann Whitney test in not‐normally distributed datasets. The normal distribution was verified according to D'Agostino and Pearson test. The two‐stage linear step‐up procedure of Benjamini, Krieger, and Yekutieli was performed as False Discovery Rate approach (Q = 1%). Finally, the lipidic dataset was processed according to a multivariate analysis using MetaboAnalyst 4.0 (http://www.metaboanalyst.ca). The lipidic dataset was imputed to remove the missing values, log (10)‐transformed and scaled according to the Pareto method.

### LION/Web Tool Lipid‐Ontology Analysis

Lipi analysis was performed on normalized data using the LION/web tool (http://www.lipidontology.com/).^[^ ^36^ ^]^ This work used the “ranking mode” analysis with a one‐tailed Welch 2 sample *t*‐test as the local statistics. Changes in lipid patterns between *HSD17B12* and scrambled siRNA cells were associated with the main branches of LION ontology and evaluated taking into consideration especially lipid function, cellular component, and physical‐chemical properties.

### Membrane Fluidity Assay

Membrane fluidity was determined using a Membrane Fluidity Kit (ab189819, Abcam), according to the manufacturer's recommendations. Cells were seeded in dark bottom 96‐well plates (7–8 × 10^3^ cells well^−1^) and three replicates were used for each experimental condition. Cells were incubated with a fluorescent lipid reagent (pyrene decanoic acid, PDA, 15 µM) supplemented with 0.08% Pluronic F127 for 1 h at 25 °C in the dark and subsequently washed in perfusion buffer three times. After incubation, cells were analyzed using the EnSpire Multimode Plate Reader (PerkinElmer) spectrophotometer by measuring the fluorescence intensity at 350 nm (excitation)/400 nm (emission) and 350 nm (excitation)/470 nm (emission) to record the formation of monomers and excimers, respectively. Quantitative monitoring of the membrane fluidity was attained by measuring the ratio of excimer (Em ≈470 nm) to monomer (Em max. ≈400 nm) fluorescence.

### Lipid Droplet Staining and Measurements

Cells were seeded in 6‐well plates (7–8 × 10^5^ cells well^−1^) containing polylysine‐coated glass coverslips (Microtech S.R.L). Cells were fixed in 4% paraformaldehyde for 10 min at room temperature, washed in cold phosphate buffer saline (PBS), and stained with a 1:200 solution of HCS LipidTOX green neutral lipid stain (Thermo Fisher, H34475) for at least 30 min at room temperature in the dark. Cell nuclei were counterstained with DRAQ5 Fluorescent Probe Solution (Thermo Fisher, #62 251). Images were acquired using a Zeiss LSM980 confocal microscope and 10 fields per each condition were imaged for analysis. Cell‐by‐cell measurements of the lipid droplet content (number of lipid droplets/cell) and dimensions in terms of average lipid droplet diameter (µm) and area (µm^2^) were performed using the ImageJ software. Measurements were performed on ≈100 cells per each experimental condition.

### Statistical Analysis

Differences between groups were analyzed using an unpaired Student's *t*‐test. Statistical significance values <0.05. * *p*‐value ≤ 0.05, ** *p*‐value ≤ 0.01, *** *p*‐value ≤ 0.001, **** *p*‐value ≤ 0.0001.

## Conflict of Interest

The authors declare no conflict of interest.

## Author Contributions

M.C. and T.M. performed the conception and design of the study. T.M. and M.A. executed the functional in vitro experiments, the analysis, and the interpretation of data. M.C., V.A., F.B., V.A.L., and G.E. conducted the bioinformatic/in silico analyses. T.M., A.M., M.C. and M.A. designed and carried out the CRISPR/Cas9 genome editing experiments, with the contribution of V.C., M.T., and L.M. In vitro targeted lipidomic assays have been carried out by M.R. and M.C., while T.M. executed the in silico lipid ontology analysis. S.C., T.M., M.M., M.A., and A.E. contributed to the validation of the genetic association in the Italian cohort of neuroblastoma patients. T.M. drafted the manuscript, with the contribution of the other authors, and under the supervision of M.C. All authors were involved in reviewing the manuscript for submission and provided feedback for revisions. All authors read and approved the final manuscript.

## Supporting information



Supporting Information

Supplemental Tables

## Data Availability

Public ATAC‐seq data are available in the NCBI Gene Expression Omnibus (GEO) under accession nos.: GSE80152, GSE138315, and GSE94824. Public DNase‐I hypersensitivity (DHS) data are available in the NCBI Gene Expression Omnibus (GEO) under accession nos.: GSE29692 and GSE32970. Public H3K27Ac ChIP‐seq data are available in the NCBI Gene Expression Omnibus (GEO) under accession no. GSE138315, GSE90683 and GSE90805. Public TF ChIP‐seq data are available in the NCBI Gene Expression Omnibus (GEO) under accession nos.: GSE138315, GSE120074, GSE80151, GSE94782, GSE94824, GSE65664, and GSE169616. Public RNA‐seq data are available in the NCBI Gene Expression Omnibus (GEO) under accession no.: GSE62564. Public eQTL data are available in the public GTEx database v8 at https://gtexportal.org/home. The in‐house generated raw ATAC‐seq, ChIP‐seq, and Hi‐C data supporting the conclusions of this article will be made available by the corresponding author upon reasonable request. The data that support the findings of this study are available from the corresponding author upon reasonable request.
