# Supplementary material for: The Non‐Coding Regulatory Variant rs2863002 at chr11p11.2 Increases Neuroblastoma Risk by Affecting HSD17B12 Expression and Lipid Metabolism
Source: Adv Sci (Weinh). 2025 Jun 17;12(33):e15181. doi: 10.1002/advs.202415181 (PMC12412506; doi:10.1002/advs.202415181)
Supplement: Supplementary file 1 — Supporting Information [file ADVS-12-e15181-s002.docx]

Supporting Information

The non-coding regulatory variant rs2863002 at chr11p11.2 increases neuroblastoma risk by affecting *HSD17B12* expression and lipid metabolism

Teresa Maiorino, Marianna Avitabile, Vincenzo Aievola, Annalaura Montella, Vito A. Lasorsa, Ferdinando Bonfiglio, Mariagrazia Cantalupo, Sueva Cantalupo, Gilda Estinto, Matilde Tirelli, Martina Morini, Martina Ardito, Alessandra Eva, Vincenza Cerbone, Lucia Mauriello, Marianna Caterino, Margherita Ruoppolo, John M. Maris, Sharon J. Diskin, Achille Iolascon, Mario Capasso^*^


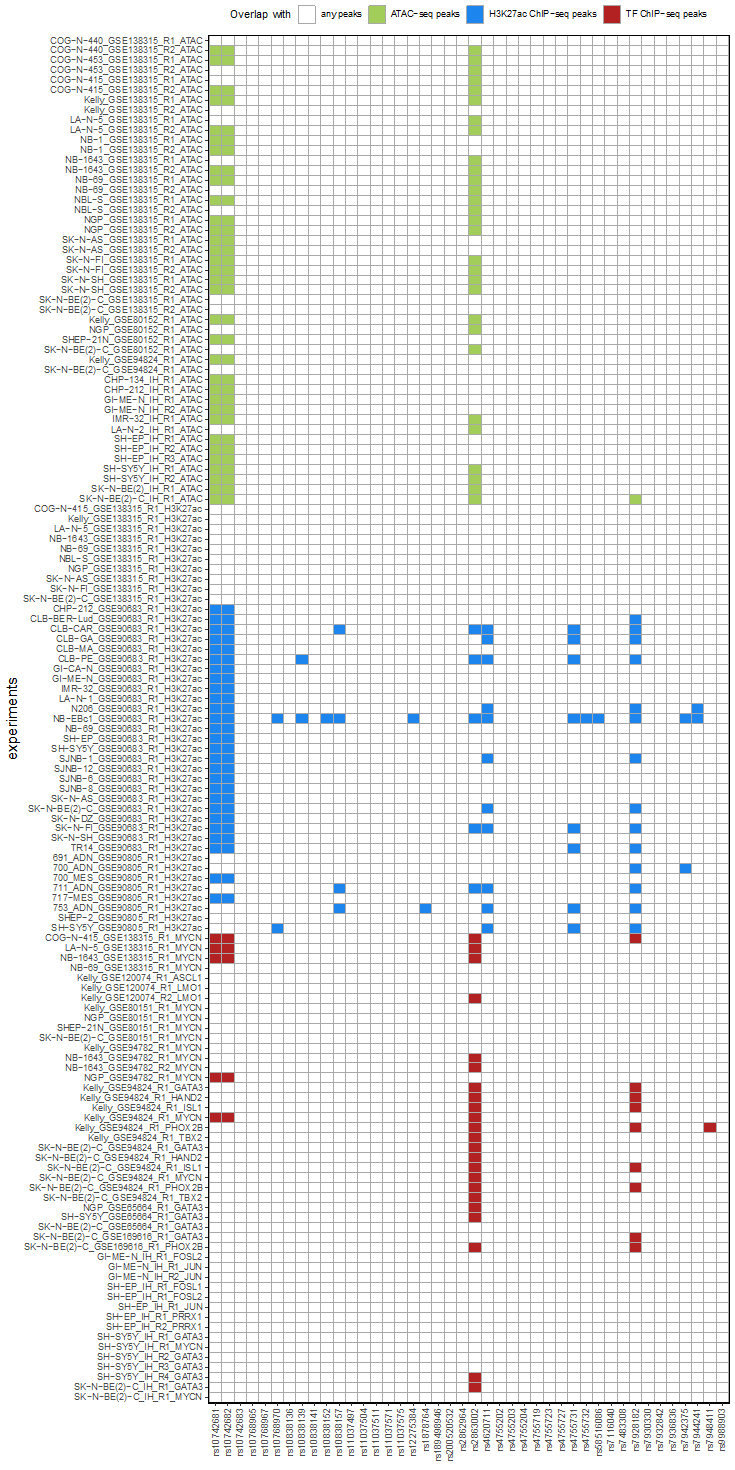


**Figure S1. Functional annotations of neuroblastoma-associated SNPs at chr11p11.2 locus from experimental data obtained in neuroblastoma cell lines.** All 42 neuroblastoma-associated SNPs selected for the chr11p11.2 risk locus (x-axis) were annotated with public (GSE138315, GSE80152, GSE90683, GSE90805, GSE120074, GSE80151, GSE94824, GSE94782, GSE65664, GSE169616) and *in-house* (IH) ATAC-seq, H3K27ac ChIP-seq and Transcription Factors (TF) ChIP-seq (MYCN, ASCL1, LMO1, GATA3, HAND2, ISL1, PHOX2B, TBX2, FOSL2, FOSL1, JUN and PRRX1) experiments in neuroblastoma cell lines (y-axis). Green, blue, and red boxes highlight SNPs overlapping with open chromatin regions (ATAC-seq records), H3K27ac marked regions, and Transcription Factors binding sites (TFBSs), respectively.


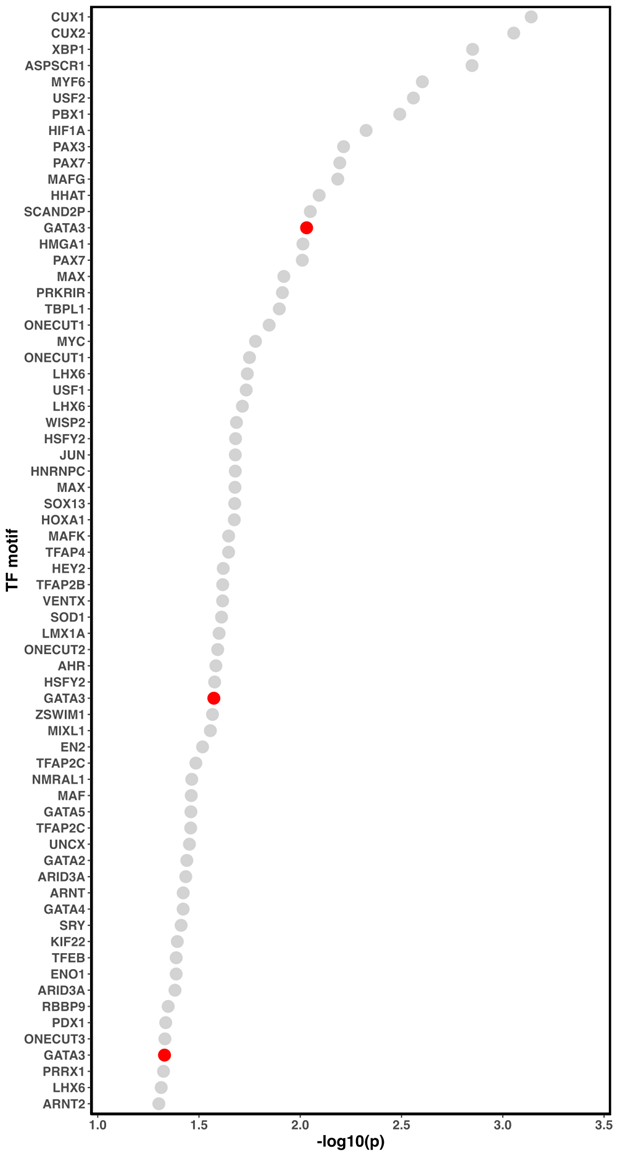


**Figure S2. Motif enrichment analysis results.** Enrichment of transcription factor (TF) motifs in a 50 bp region centered on rs2863002 at chr11:43714768 (hg19/GRCh37) was calculated using PWMEnrich. P values (p) ≤ 0.05 were considered significant. Red dots indicate the enrichment of motifs interacting with TF whose binding was also confirmed by ChIP-seq experiments in neuroblastoma cell lines.


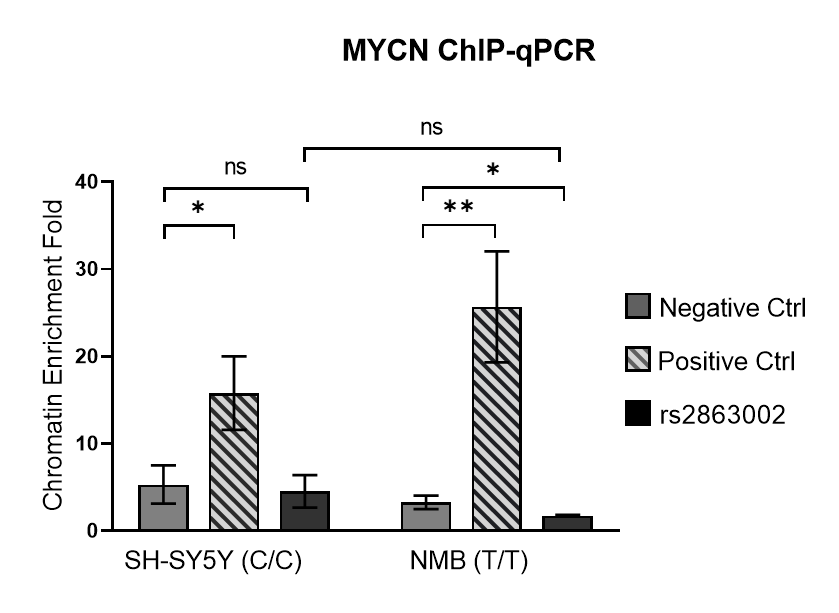


**Figure S3. Results of the ChIP qPCR experiments for the TF MYCN performed in neuroblastoma cell lines.** Chromatin fold enrichment was obtained by immunoprecipitation with the MYCN antibody (Abcam, ab227822) in ChIP qPCR experiments carried out in SH-SY5Y and NMB neuroblastoma cells, carrying the C/C and the T/T rs2863002 genotypes respectively. We report the chromatin fold enrichment obtained for a negative (chr2:80,521,594-80,524,257) and a positive (chr5:170,814,842-170,815,848) DNA control region for MYCN binding, respectively in dark grey and light dashed gray; in black is the chromatin fold enrichment obtained for the genomic region of rs2863002. Enrichment measurements are folded on Rabbit IgG and represent the mean ±SD from three independent ChIP qPCR experiments. ns not significant; * p-value < 0.05; ** p-value < 0.01; *** p-value < 0.001. P-values were calculated by t-test.

**
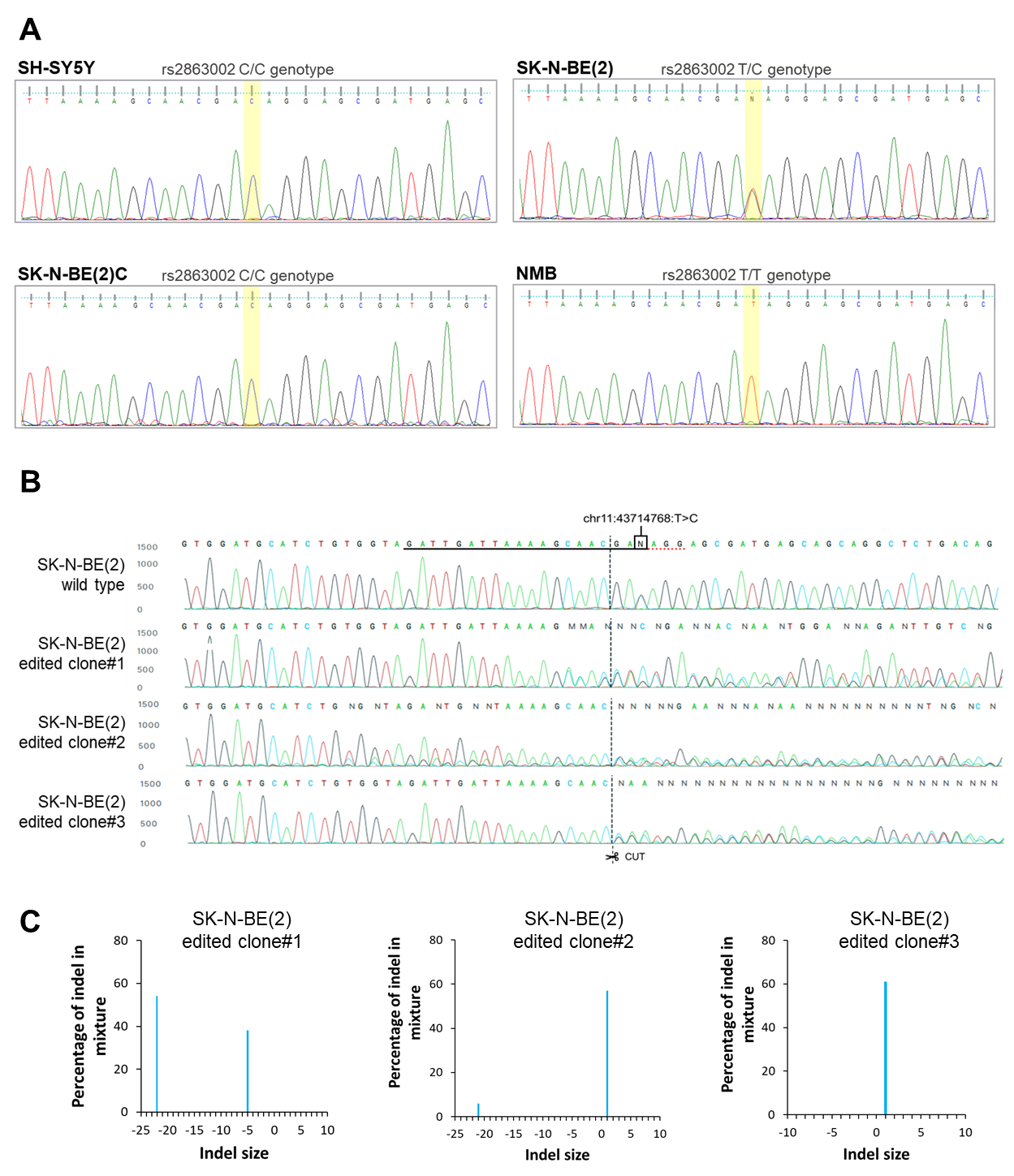
**

**Figure S4.** **Genome editing of rs2863002 at 11p11.2 locus through CRISPR/Cas9 experiments in SK-N-BE(2) neuroblastoma cell line. (A)** Sanger sequencing electropherograms showing part of a 388pb amplicon of the genomic region containing rs2863002 (chr11:43714768, hg19/GRCh37) obtained from the SH-SY5Y, SK-N-BE(2)C, SK-N-BE(2), and NMB neuroblastoma cell lines. The yellow column highlights rs2863002T>C position, and the various SNP genotypes are reported for each cell line. **(B)** Sanger sequencing traces showing the Cas9 cutting site in proximity of chr11:43714768:rs2863002T>C variant (vertical dotted line) and subsequent waveform decomposition in edited clones #1, #2, and #3, compared to the SK-N-BE(2) wild type cell line (top). Black line: gRNA sequence; red dotted line: PAM sequence; black square: rs2863002 genomic location. **(C)** Bar plots representing the INDEL (insertion/deletions) distribution in SK-N-BE(2) edited clones #1 (left), #2 (middle) and #3 (right). The x-axis reports the size of the INDELs in base pairs.


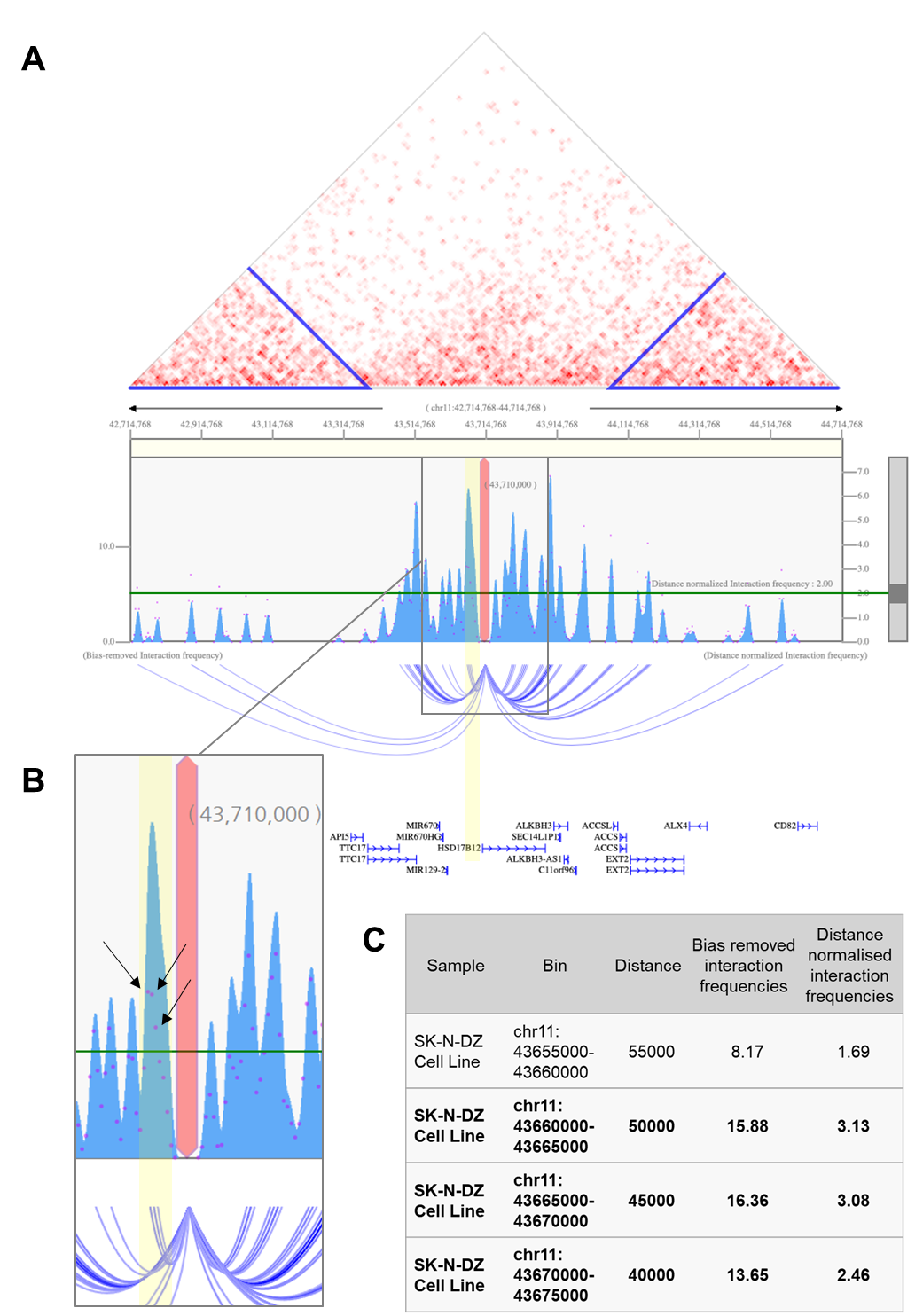


**Figure S5.** **Physical interaction between rs2863002 and *HSD17B12* promoter region from public Hi-C data in SK-N-DZ neuroblastoma cell line.** **(A)** Plot of Hi-C data from SK-N-DZ cell line (3DIV web tool) showing the genomic regions that interact with rs2863002. The interaction matrix is centered on the SNP position at chr11:43714768 (hg19/GRCh37) and extended of 1Mb up- and down-stream. From top to bottom the image shows: the blue-bordered triangles that represent the Topologically Associated Domains (TADs); the distance normalized interaction frequency, as a measure of the strength of interactions; the arcs, representing the predicted bindings between rs2863002 genomic position and the promoters of the surrounding genes; the NCBI RefSeq genes. The pink column represents the rs2863002 genomic location while the yellow rectangle highlights the interaction involving the *HSD17B12* promoter region. **(B)** Zoom-in panel showing in more detail the region surrounding rs2863002. The black arrows highlight the most relevant chromatin interaction signals (distance normalized interaction frequency >2). **(C)** Table reporting the numerical data as downloaded from 3DIV tool concerning the Hi-C interactions of rs2863002 with genomic regions located near the promoter of *HSD17B12*. The most relevant interactions are highlighted in bold, corresponding to those pointed out by the arrows in panel B.


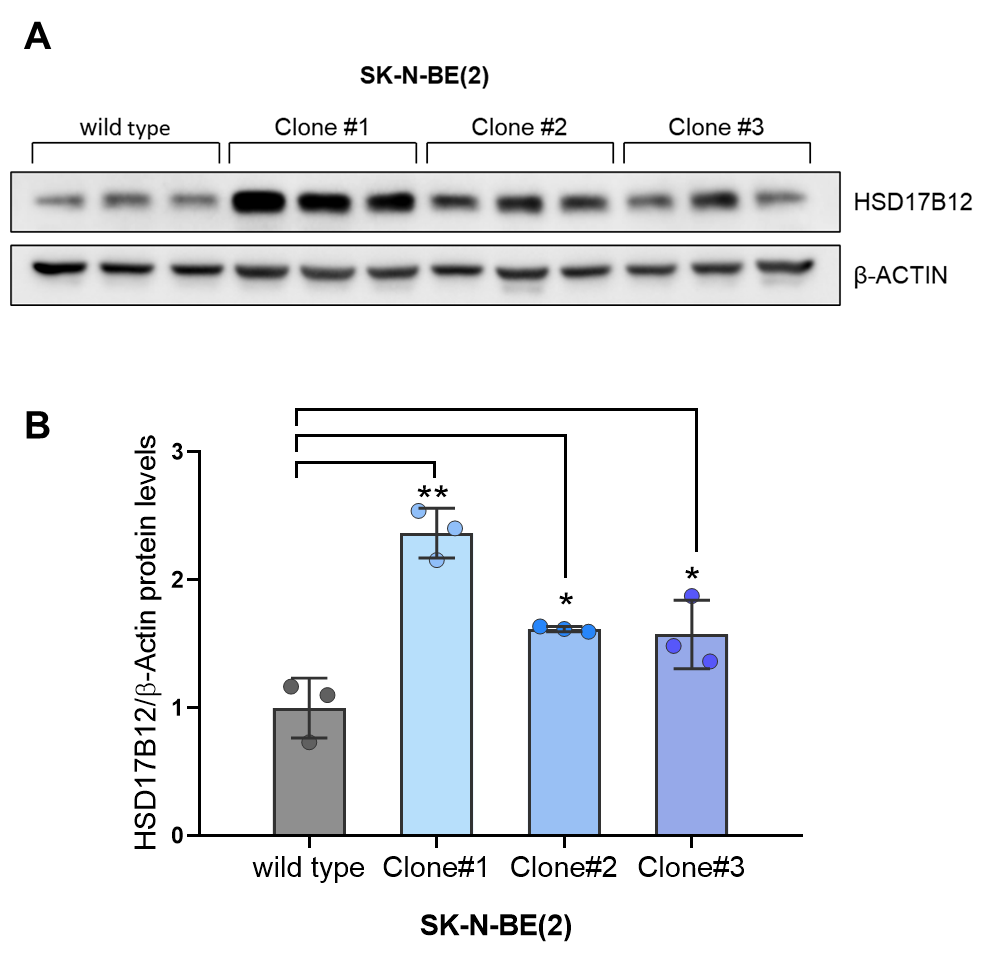


**Figure S6. HSD17B12 protein expression in SK-N-BE(2) wild-type and CRISPR/Cas9 edited clones.**

**(A)** Western blot image of HSD17B12 protein expression in triplicate samples of SK-N-BE(2) wild-type cells and three CRISPR/Cas9-edited clones at rs2863002. Triplicates were derived from independent cultures and processed concurrently on the same gel for transfer and film development. β-Actin protein level was used as the loading control. **(B)** Densitometric analysis of HSD17B12 protein expression normalized to β-actin in wild-type and three CRISPR-edited SK-N-BE(2) clones (Clone#1–3). Data are presented as mean ± SD (n = 3 biological replicates), with dots representing single measurements. Statistical significance was determined using t-test. * p-value < 0.05; ** p-value < 0.01 vs. wild-type.


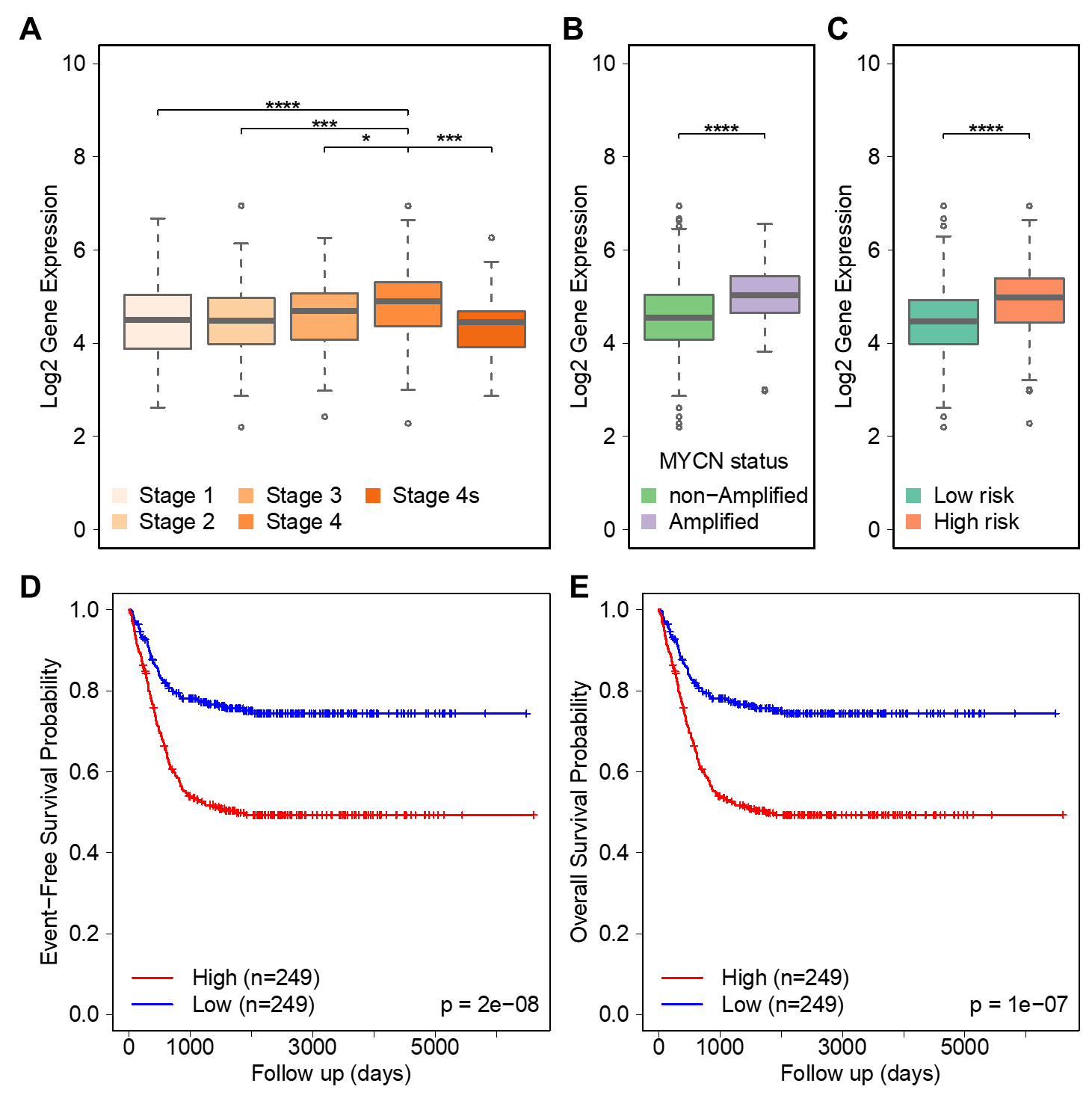


**Figure S7. High *HSD17B12* expression correlates with unfavorable clinical markers and poor prognosis in neuroblastoma patients. (A-C)** Box plots showing the mRNA expression of *HSD17B12* in the GSE62564 neuroblastoma patients’ dataset from R2 Genomics (Tumor Neuroblastoma - SEQC - 498 - RPM - seqcnb1). Patients are stratified according to stage classification (A), *MYCN* amplification status (B), and risk groups (C). The dark line in the middle of the box plots shows the median expression value, while the bottom and the top of each box indicate the 25^th^ and the 75^th^ percentile, respectively. The T-bars that extend from the boxes indicate the minimum and maximum values. * p-value < 0.05; ** p-value < 0.01; *** p-value < 0.001; **** p-value <0.0001. P-values obtained by two-tailed T-test. **(D-E)** Kaplan-Meier curves showing event-free (D) and overall (E) survival for neuroblastoma patients (GSE62564) grouped according to *HSD17B12* expression levels. Log-rank p-values are shown.


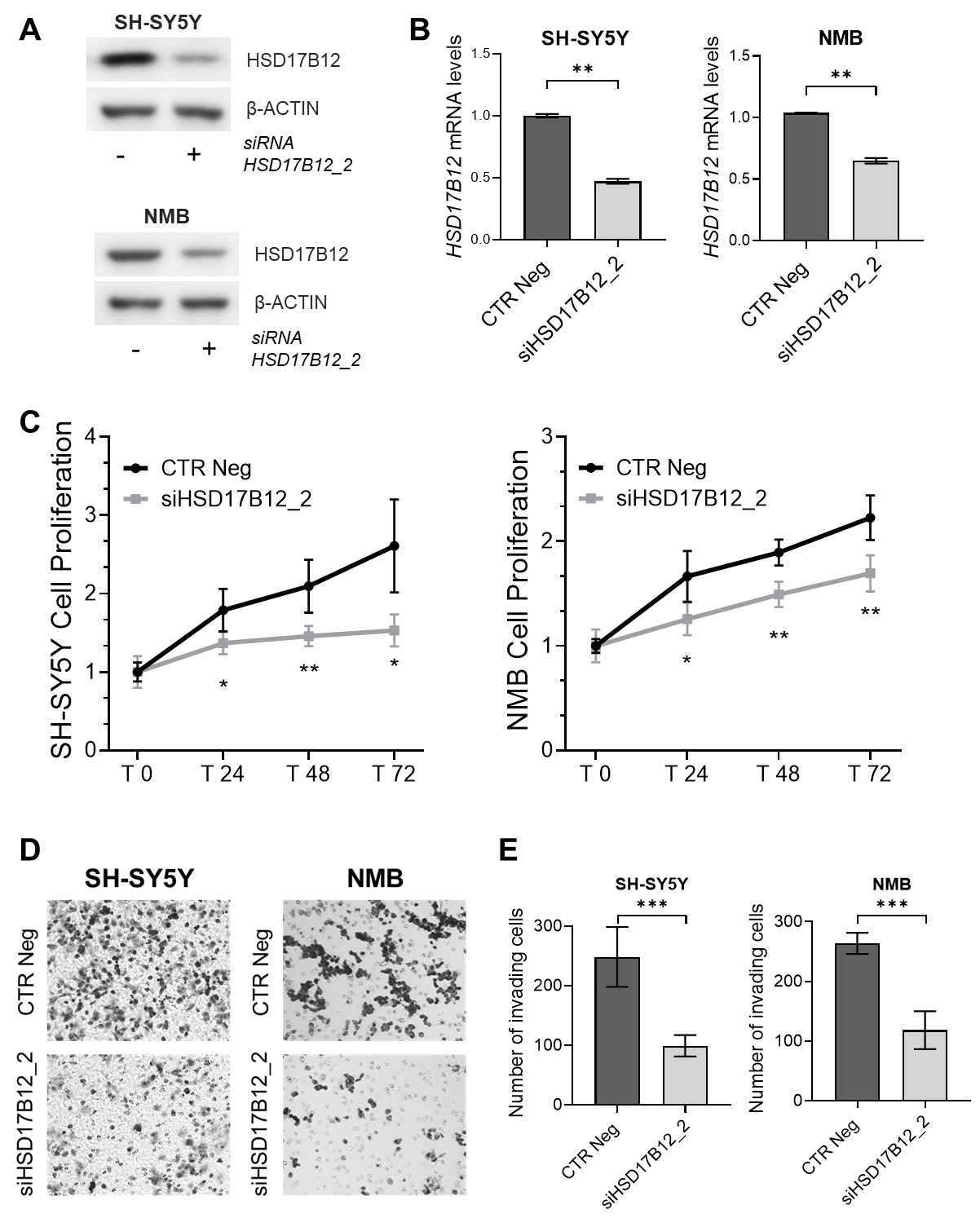


**Figure S8.** **Silencing of *HSD17B12* with 4-pooled siRNA and its pro-tumoral effects on cell proliferation and invasiveness.** **(A-B)** The efficiency of *HSD17B12* silencing was measured by western blot (A) and qRT-PCR (B) in SH-SY5Y and NMB neuroblastoma cell lines 72h post siRNA transfection by using ON-TARGETplus Human HSD17B12 SMARTpool siRNAs. Data represent the mean ±SD from three independent experiments. **(C)** Assessment of cell proliferation in SH-SY5Y (left) and NMB (right) cell lines after silencing of *HSD17B12*. Cell viability measurements were performed using MTT assays at 0, 24, 48, and 72h post siRNA transfection. Data shown are the mean ±SD from two independent MTT experiments, with six technical replicates for each experimental point. **(D)** Representative images of trans-well invasion assays performed in SH-SY5Y and NMB cell lines after silencing of *HSD17B12*. The cells have been fixed after migrating over-night and stained with Hematoxylin & Eosin (Sigma-Aldrich). Invading cells were counted using the Leica Application Suite/AF software and DMI4000B microscope (Leica Microsystem). Images were acquired using 5x to 20x magnification. **(E)** Number of invasive cells as measured in trans-well invasion assays performed in SH-SY5Y and NMB after silencing of *HSD17B12*. Data represent the mean ±SD from two independent experiments. * p-value < 0.05; ** p-value < 0.01; *** p-value < 0.001 compared with control conditions (ON-TARGETplus Non-Targeting Control siRNA #1). P-values obtained by t-test.


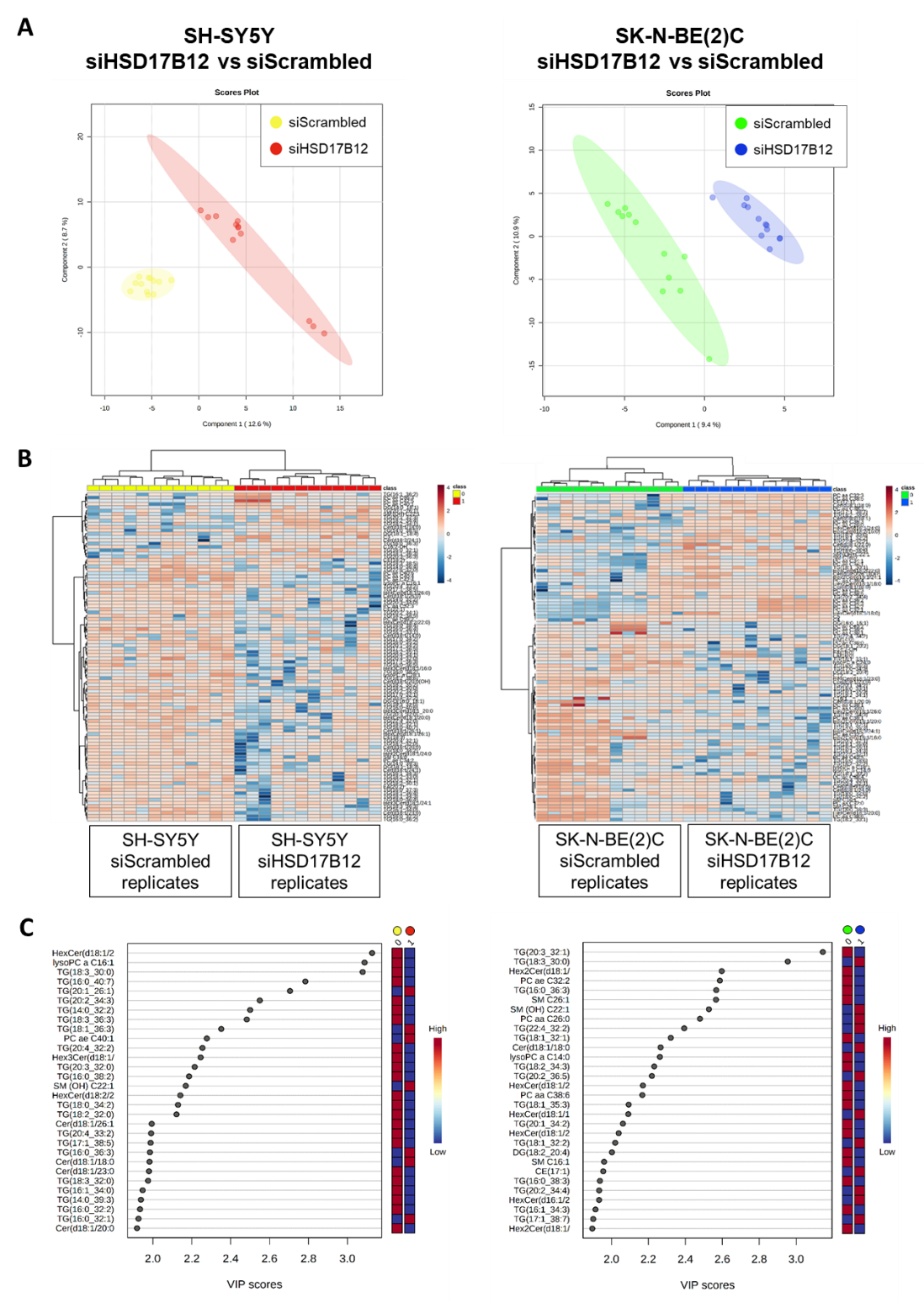


**Figure S9.** **Lipid profiling of neuroblastoma cells after *HSD17B12* silencing. (A)** Supervised partial least squares-discriminant analysis (PLS-DA) score plots in SH-SY5Y (left) and SK-N-BE(2)C (right) cells silenced for *HDS17B12* compared with siRNA Scrambled controls. Four biological and three technical replicates for each of them have been analysed for every experimental condition. **(B)** Heatmaps of the auto-scaled mean concentrations of significantly altered lipids (p < 0.05) in SH-SY5Y (left) and SK-N-BE(2)C (right) after *HSD17B12* silencing (0 scrambled, 1 *HSD17B12* siRNA). The heatmap color code represents the relative lipid abundance. The concentrations of the metabolites were imputed, log(2) transformed, and Pareto scaled. **(C)** Discriminant lipidic features according to the variable importance on projection (VIP) in SH-SY5Y (left) and SK-N-BE(2)C (right) silenced cells. The top 30 important lipids (VIP score ≥ 2.0) were summarized according to their VIP score value. The intensity of the colored boxes indicates the relative lipid abundance in each group (0 scrambled, 1 *HSD17B12* siRNA).


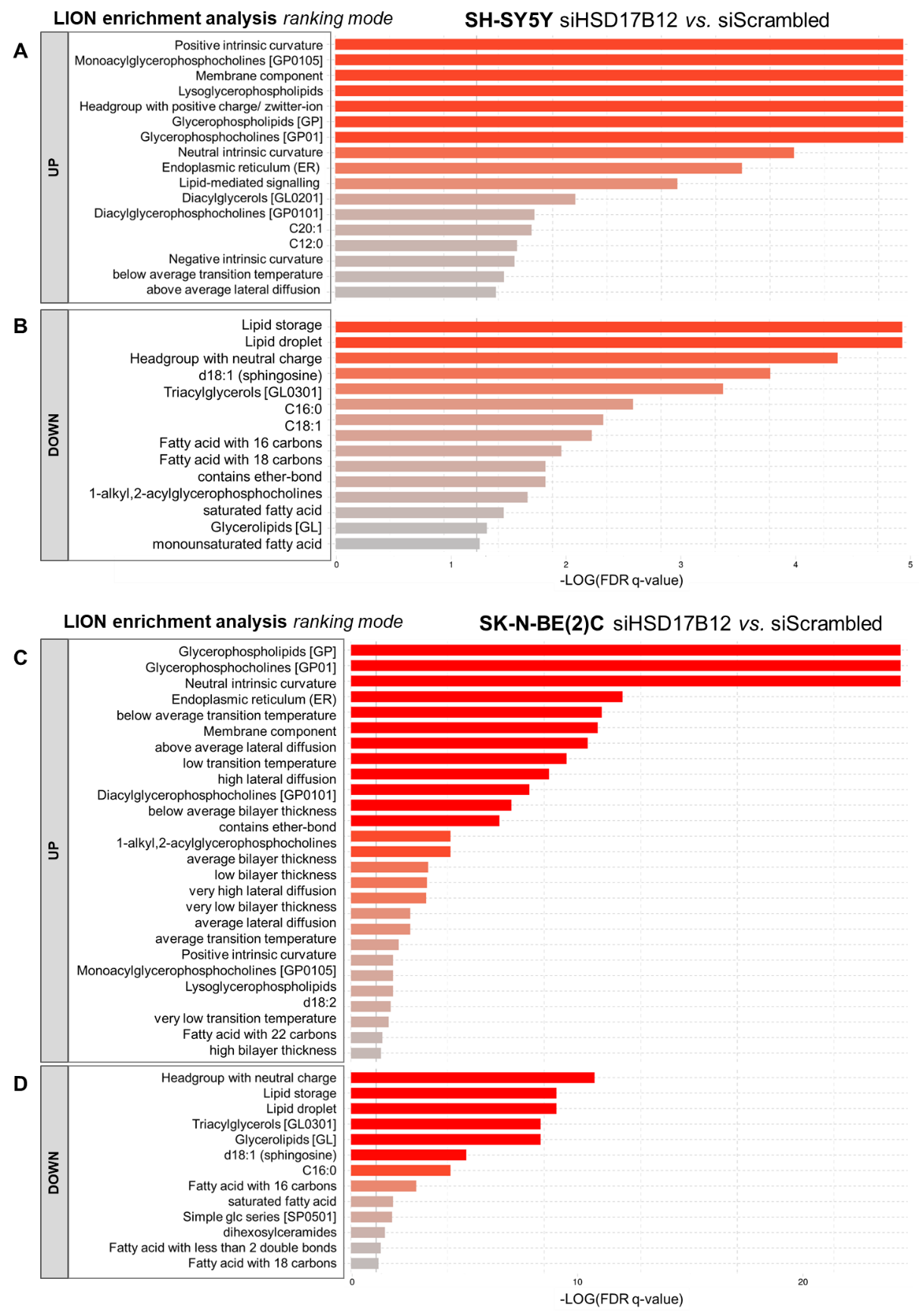


**Figure S10. Enrichment analysis of neuroblastoma cells silenced for *HSD17B12* using LION web tool.** Enrichment analysis of significantly altered lipid species in SH-SY5Y **(A-B)** and SK-N-BE(2)C **(C-D)** after *HSD17B12* silencing. The dotted line indicates the cut-off value of significant enrichments (q < 0.05). Bar length is related to the entity of enrichment (-log10 q-values, corrected for false discovery rate, FDR).


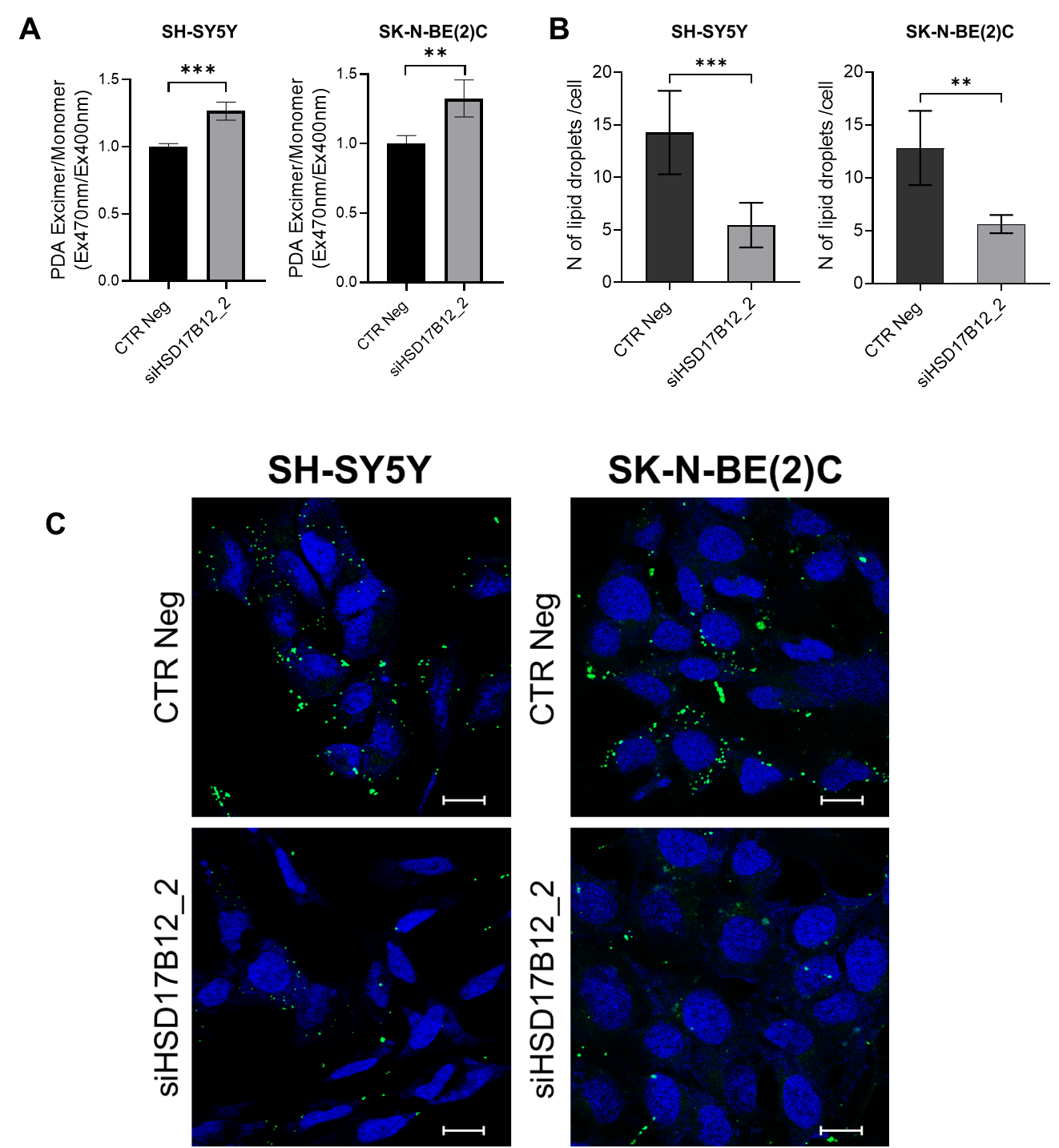


**Figure S11.** **Silencing of *HSD17B12* with 4-pooled siRNA and its effects on cell membrane fluidity and lipid droplet number. (A)** Membrane fluidity was assessed by measuring the ratio of pyrene-decanoic acid (PDA) excimer to monomer fluorescence in SH-SY5Y (left) and SK-N-BE(2)C (right) cells after silencing of *HSD17B12* with ON-TARGETplus Human HSD17B12 SMARTpool siRNAs. Fluorescence was evaluated at 400nm for monomers and 470nm for excimers. Data represent the mean ± SD of the measurements compared with control conditions (ON-TARGETplus Non-Targeting Control siRNA #1) from two independent experiments performed in duplicate. **(B)** Quantification of lipid droplet number obtained through cell-by-cell measurements in SH-SY5Y (left) and SK-N-BE(2)C (right) cells after silencing of *HSD17B12*. Data represent the mean number ± SD of lipid droplets per cell; measurements have been performed on a mean number of 100 cells per experimental condition. **(C)** Representative confocal images of neutral lipid staining by LipidTOX^TM^ Green (green) in SH-SY5Y and SK-N-BE(2)C cells after silencing of *HSD17B12*. Nuclei were counterstained with DRAQ5™ Fluorescent Probe Solution (blue) and Images were acquired using a Zeiss LSM980 confocal microscope. Scale bar 20µM. * p-value < 0.05; ** p-value < 0.01. P-values were calculated by t-test.


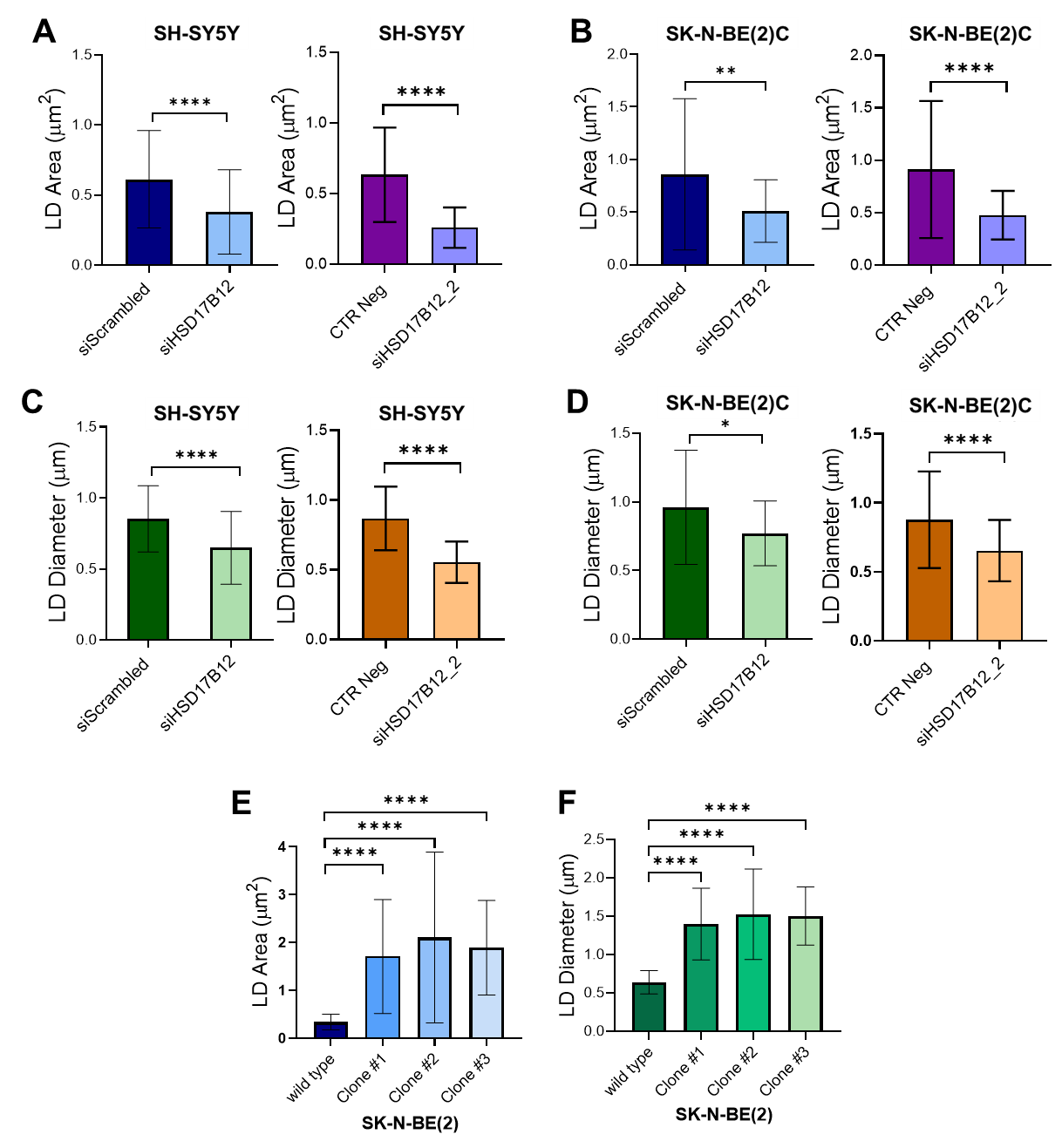


**Figure S12. Effects of *HSD17B12* expression alterations on lipid droplets size.** **(A-D)** Mean cell-by-cell measurements of lipid droplets area (µm^2^) (A-B) and diameter (µm) (C-D) in SH-SY5Y and SK-N-BE(2)C after *HSD17B12* silencing. Gene Silencing was obtained through two distinct siRNA approaches: a pool of 3 different siRNA by Origene (labeled as siHSD17B12) vs a control condition (labeled as siScrambled), and a pool of 4 different siRNA by Horizon (labeled as siHSD17B12_2) vs a control condition (labeled as CTR Neg). **(E-F)** Mean cell-by-cell measurements of lipid droplets area (µm^2^) (E) and diameter (µm) (F) in SK-N-BE(2) wild-type and CRISPR/Cas9-edited clones (clone #1, #2 and #3). Measurements have been performed on approximately 100 cells per each experimental condition. Data represent the mean ± SD of the measurements and statistical significance has been calculated by t-test student vs control conditions. * p-value ≤ 0.05; ** p-value ≤ 0.01; **** p-value ≤0.0001.
